# Parkinson's disease, aging and adult neurogenesis: Wnt/β‐catenin signalling as the key to unlock the mystery of endogenous brain repair

**DOI:** 10.1111/acel.13101

**Published:** 2020-02-12

**Authors:** Bianca Marchetti, Cataldo Tirolo, Francesca L'Episcopo, Salvatore Caniglia, Nunzio Testa, Jayden A. Smith, Stefano Pluchino, Maria F. Serapide

**Affiliations:** ^1^ Department of Biomedical and Biotechnological Sciences (BIOMETEC) Pharmacology and Physiology Sections Medical School University of Catania Catania Italy; ^2^ Neuropharmacology Section OASI Research Institute‐IRCCS Troina Italy; ^3^ Department of Clinical Neurosciences and NIHR Biomedical Research Centre University of Cambridge Cambridge UK

**Keywords:** adult neurogenesis, aging, neuroinflammation, Parkinson's disease, plasticity/self‐repair, Wnt/β‐catenin signalling

## Abstract

A common hallmark of age‐dependent neurodegenerative diseases is an impairment of adult neurogenesis. *Wingless‐type mouse mammary tumor virus integration site (Wnt)/β‐catenin* (WβC) signalling is a vital pathway for dopaminergic (DAergic) neurogenesis and an essential signalling system during embryonic development and aging, the most critical risk factor for Parkinson's disease (PD). To date, there is no known cause or cure for PD. Here we focus on the potential to reawaken the impaired neurogenic niches to rejuvenate and repair the aged PD brain. Specifically, we highlight WβC*‐*signalling in the plasticity of the subventricular zone (SVZ), the largest germinal region in the mature brain innervated by nigrostriatal DAergic terminals, and the mesencephalic aqueduct‐periventricular region (Aq‐PVR) Wnt‐sensitive niche, which is in proximity to the SNpc and harbors neural stem progenitor cells (NSCs) with DAergic potential. The hallmark of the WβC pathway is the cytosolic accumulation of β‐catenin, which enters the nucleus and associates with T cell factor/lymphoid enhancer binding factor (TCF/LEF) transcription factors, leading to the transcription of *Wnt* target genes. Here, we underscore the dynamic interplay between DAergic innervation and astroglial‐derived factors regulating WβC‐dependent transcription of key genes orchestrating NSC proliferation, survival, migration and differentiation. Aging, inflammation and oxidative stress synergize with neurotoxin exposure in “turning off” the WβC neurogenic switch via down‐regulation of the nuclear factor erythroid‐2‐related factor 2/Wnt‐regulated signalosome, a key player in the maintenance of antioxidant self‐defense mechanisms and NSC homeostasis. Harnessing WβC‐signalling in the aged PD brain can thus restore neurogenesis, rejuvenate the microenvironment, and promote neurorescue and regeneration.

## Graphical abstract

Harnessing WβC signalling activation in the aged, inflamed PD brain. A “Wnt‐on” neurorestoration program instructed by grafted NSCs and a panel of pharmacological treatments rescuing the impaired neurogenic niches is illustrated. With age, the inflamed microenvironment coupled to dysfunctional astrocyte–microglia interactions and environmental toxin exposure (MPTP) inhibit active Wnt signalling (“Wnt‐off” condition), leading to exacerbation of inflammation and inhibition of Wnt‐dependent proregenerative/self‐repair potential, with harmful consequences for SVZ and Aq‐PVR niches, mDA neuron survival and repair from MPTP injury. The ability of NSC grafts, NSC‐derived astrocytes and endogenous astrocytes to switch the inflammatory/Wnt‐genetic cascade via astrocyte–neuron and astrocyte–microglia crosstalk both at the SNpc and at the Aq‐PVR DA niche levels is illustrated. Reciprocally, astrocyte‐derived Wnt1 further influences both exogenous and endogenous NSCs and reduces microglia pro‐inflammatory status, thus favouring beneficial effects for an overall TH neurorescue (“Wnt on”) program.

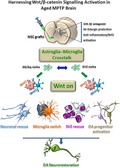

**Abbreviations**
3V: third ventricle
6‐OHDA: 6‐hydroxydopamine
APC: adenomatous polyposis coli
AQ: amodiaquine
Aq‐PVR: aqueduct‐periventricular region
AR: AR‐AO14418
BDNF: brain‐derived neurotrophic factor
BrdU: bromodeoxyuridine
CHIR: CHIR99021
CK1α: casein kinase 1α
CRD: cysteine‐rich domain
CSF: cerebrospinal fluid
DA: dopamine
DAergic: dopaminergic
DAT: dopamine transporter
DCX: doublecortin
DG: dentate gyrus
Dkk: Dickkopf
Dvl: Dishevelled
EGF: epidermal growth factor
EGFR: epidermal growth factor receptor
En: engrailed
Fzd: Frizzled
GBA1: β‐glucocerebrosidase 1
GFAP: glial fibrillary acidic protein
GSK‐3β: glycogen synthase kinase 3β
HLY78: 4‐ethyl‐5‐methyl‐5,6‐dihydro‐[1,3]dioxolo[4,5‐j]phenanthridine
Hmox1: heme oxygenase 1
IBA1: ionized calcium‐binding adapter molecule 1
icv: intracerebroventricular
iNOS: inducible nitric oxide synthase
iPSC: induced pluripotent stem cell
L‐DOPA: levodopa
LEF: lymphoid enhancer binding factor
LGR: leucine‐rich repeat‐containing G‐protein coupled receptors
LMX1B: LIM homeobox transcription factor 1β
LRP: low‐density lipoprotein receptor‐related protein
LRRK2: leucine‐rich repeat kinase 2
MAP2a: microtubule‐associated protein 2a
mNSC: midbrain Aq‐PVR‐NSC
MPP^+^: 1‐methyl‐4‐phenylpyridinium
MPTP: 1‐methyl‐4‐phenyl‐1,2,3,6‐tetrahydropyridine
mtDNA: mitochondrial DNA
ND: neurodegenerative disorder
Nrf2: nuclear factor erythroid‐2‐related factor 2
NSAID: non‐steroidal anti‐inflammatory drug
NSC: neural stem cell
Nurr1: nuclear receptor‐related factor 1
OB: olfactory bulb
PCP: planar cell polarity
PD: Parkinson's disease
PDD: Parkinson's disease with dementia
PHOX: phagocyte oxidase
PI3K: phosphoinositide 3‐kinase
PINK1: PTEN‐induced putative kinase
PP2A: protein phosphatase‐2A
PTX: paclitaxel
RMS: rostral migratory stream
RNF43: ring finger protein 43
RNS: reactive nitrogen species
ROR: receptor tyrosine kinase‐like orphan receptor
ROS: reactive oxygen species
Rspo: R‐spondin
RYK: receptor‐like tyrosine kinase
SAMP8: senescence associated mouse prone 8
sFRP: secreted Fzd‐related protein
SGZ: subgranular zone
SN: substantia nigra
SNpc: substantia nigra pars compacta
Str: striatum
SVZ: subventricular zone
TAP: transit‐amplifying progenitor cells
TCF: T cell factor
Tfam: transcription factor A
TH: tyrosine hydroxylase
TNFα: tumor necrosis factor α
VM: ventral midbrain
VTA: ventral tegmental area
WIF: Wnt inhibitory factor
WIP1: wild‐type p53‐induced phosphatase 1
Wnt: wingless‐type mouse mammary tumor virus integration site
WβC: Wnt/β‐catenin
ZNRF3: zinc and ring finger 3
α‐syn: α‐synuclein

## 1 INTRODUCTION

Aging is the leading risk factor for Parkinson's disease (PD), the second most diagnosed neurodegenerative disorder (ND), affecting almost 1% of the population over age 60 (Blauwendraat et al., 2019). Typical neuropathological hallmarks of PD include the selective loss of dopaminergic (DAergic) cell bodies in the subtantia nigra pars compacta (SNpc) in the mesencephalon and their projections to the striatum (Str), with consequent depletion of striatal dopamine (DA), the deposition of cytoplasmic fibrillary inclusions (Lewy bodies) containing ubiquitin and α‐synuclein (α‐syn), and astroglial activation (Schapira et al., 2014). The cardinal motor signs of PD include a combination of bradykinesia, postural instability, and resting tremor. Non‐motor symptoms including hyposmia, cognitive dysfunction, and sleep and mental health disorders, often precede and/or accompany PD onset and progression, but the underlying pathological alterations in the brain are not fully understood (Reichmann et al., 2016; Schapira, Chaudhuri, & Jenner, 2017).

PD is the fastest growing ND, and because the world's population is aging the number of individuals affected is expected to grow exponentially: the number of people with PD is forecast to double from 6.9 million in 2015 to 14.2 million in 2040 (Dorsey & Bloem, 2018). Currently, most PD symptoms appear when ≥70% of the DAergic terminals are degenerated in the Str and more than half of the DA synthesizing neurons are lost in the SNpc, therefore early detection and intervention is crucial for effective neuroprotective treatment intended to prevent the degeneration of DAergic neurons and, ultimately, PD pathogenesis (Jankovic, 2019). To date, there are no effective treatments that can stop or reverse the neurodegeneration process in PD and current treatments rely on DAergic drugs, including levodopa (L‐DOPA) and DAergic agonists, which only temporarily alleviate motor symptoms (Obeso et al., 2017; Olanow, 2019; Olanow & Schapira, 2013).

Significantly, approximately 10% of PD cases can be directly attributed to genetic factors, associated with mutations in genes including *α*‐synuclein (*SNCA*), E3 ubiquitin‐protein ligase parkin (*PRKN*), ubiquitin C‐terminal hydrolase L1 (*UCHL1*), PTEN‐induced putative kinase (*PINK1*), DJ‐1 (*PARK7*), leucine‐rich‐repeat kinase 2 (*LRRK2*), vacuolar protein sorting 35 homolog gene (*VPS35*), and β‐glucocerebrosidase 1 (*GBA1*), linked to autosomal dominant late‐onset. In contrast, the etiology of the vast majority (up to 90%) of so called “idiopathic” PD cases is multifactorial, likely arising from a combination of polygenic inheritance and environmental exposures, with gene‐environment interactions playing a decisive role in PD onset and/or progression (Blauwendraat et al., 2019; Cannon & Greenamyre, 2013; Dzamko, Geczy, & Halliday, 2015; Guttuso, Andrzejewski, Lichter, & Andersen, 2019; Langston, 2017; Lastres‐Becker et al., 2012; L’Episcopo, Tirolo, Testa et al. 2010a; Marchetti and Abbracchio, 2005).

Aging represents the most crucial event, linking increased inflammation and oxidative stress to mitochondrial dysfunction and dysregulation of lysosomal, proteosomal and autophagic functions, likely contributing to the chronic neuronal deterioration in the PD brain (Boger, Granholm, McGinty, & Middaugh, 2010; Dzamko et al., 2015; Giguère, Burke Nanni, & Trudeau, 2018; Marchetti & Abbracchio, 2005; McGeer & McGeer, 2008; Nguyen et al., 2019; Surmeier, 2018). Hence, with advancing age, the nigrostriatal DAergic system progressively declines and the adaptive/compensatory DAergic potential gradually fails, leading to the slow but inexorable nigrostriatal degeneration of PD with the late appearance of clinical signs (Bezard & Gross, 1998; Collier et al., 2007; de la Fuente‐Fernández et al., 2011; Hindle, 2010; Hornykiewicz, 1993; Obeso et al., 2017).

**Figure 1:**
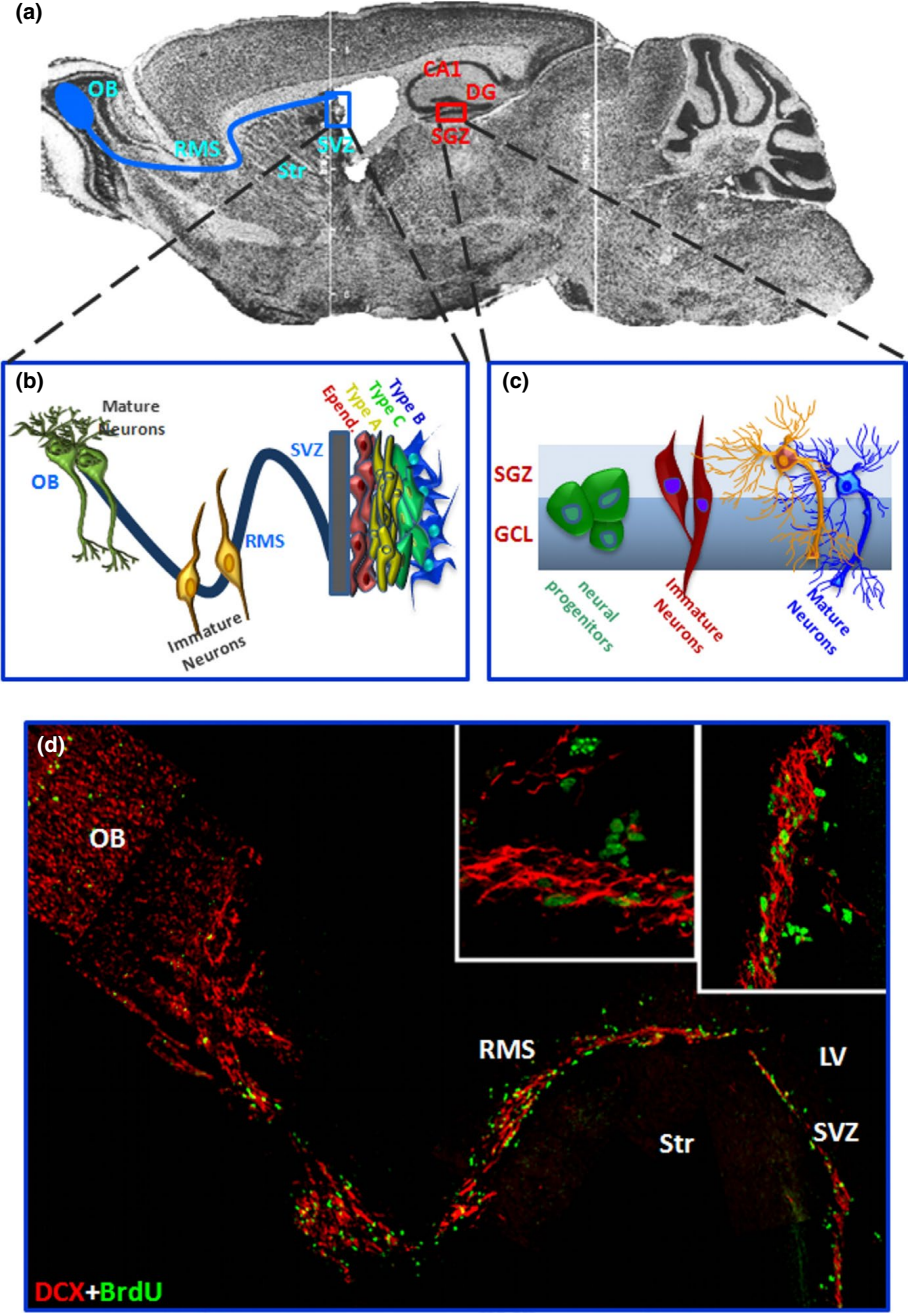
The subventricular (SVZ) and the subgranular (SGZ) zones of the adult rodent brain. (a) Sagittal brain section showing the subventricular zone (SVZ) lining the lateral ventricles (LV) and the adjacent striatum (Str), and the hippocampal subgranular zone (SGZ). In blue the trajectory of migrating neuroblasts along the rostral migratory stream (RMS) reaching the olfactory bulb (OB); in red, the CA1 field of the hippocampus and the SGZ in the dentate gyrus (DG) are shown. (b, c) Schematic representation of the neurogenic regions in the adult brain. The SVZ niche (b) composed of SVZ astrocytes (type B1 cells), rapidly proliferating (type C) cells, migrating neuroblast (type A cells), which migrate through the RMS to the OB, and ependymal cells (type E cells) (Doetsch et al., 1999, 1997). In the SGZ, radial glia‐like precursors (RGLs) within the SGZ serve as one type of quiescent NSCs (type 1 cells) and continuously give rise to both DG neurons and astrocytes (Bonaguidi et al., 2011). Mature granule neurons then migrate into the granule cell layer (GCL). (d) A sagittal reconstruction of dual immunofluorescent stained images by confocal laser scanning microscopy. SVZ‐migrating doublecortin‐positive (DCX^+^) neuroblasts in red, and dividing, bromodeoxyuridine‐positive (BrdU^+^) NSCs in green, are seen forming chains traveling along the RMS to the OB where they become granular and periglomerular interneurons involved in odor discrimination. Magnifications of DCX^+^/BrdU^+^ NSCs are shown in the boxed areas

A cardinal feature of aging and PD is the diminishment of adult neurogenesis, an active process present in most mammalian species including humans whereby new neurons are generated throughout life from neural stem/progenitor cell (NSC) activation within their specialized neurogenic niches (Apple, Solano‐Fonseca, & Kokovay, 2017; Bond, Ming, & Song, 2015; Chandel, Jasper, Ho, & Passegué, 2016; Gage, 2000; Ming & Song, 2005; Winner, Desplats, et al., 2009a). NSCs are self‐renewing, multipotent and undifferentiated precursor cells that have the ability to differentiate into glial and neuronal lineages. In physiological conditions, only two specific areas, i.e. the subventricular zone (SVZ) lining the lateral ventricles and the subgranular zone (SGZ) of the dentate gyrus (DG) in the hippocampus (Figure 1), can produce new neurons, with the potential to support odour discrimination, spatial learning, and contextual memory capabilities (Alvarez‐Builla, Garcia‐Verdugo, & Tramontin, 2001; Eriksson et al., 1998; Fuentealba, Obernier, & Alvarez‐Buylla, 2012; Gage, 2000; Spalding et al., 2013). In both regions slowly dividing quiescent NSCs give rise to activated NSCs, which rapidly differentiate into transit‐amplifying progenitor cells (TAPs) and subsequently into immature neurons (Encinas et al., 2011; Obernier et al., 2018). Early impairment of SVZ and hippocampal SGZ neurogenesis is implicated in PD‐associated pre‐motor symptoms, which may in turn contribute critically to the disease process (Agnihotri et al., 2019; Lim, Bang, & Choi, 2018; Titova et al., 2017; Winner & Winkler, 2015).

Adult neurogenesis is activated by various brain injuries, generating new neurons that migrate along the blood vessels toward an injured area where they may repair damaged tissue (Kojima et al., 2010; Yamashita et al., 2006). NSCs residing in the adult human SVZ may generate neurons that migrate to the Str, rather than into the olfactory bulb (Ernst et al., 2014). Otherwise “dormant” NSC subpopulations can be activated under specific CNS injuries and via cell‐specific signalling mechanisms (Llorenz‐Bobadilla et al., 2015). After pathological stimulation, neuroprogenitors can be activated in regions otherwise considered to be non‐neurogenic, such as the Str (Bedard, Cossette, Levesque, & Parent, 2002; Dayer, Cleaver, Abouantoun, & Cameron, 2005; Inta, Cameron, & Gass, 2015; Luzzati et al., 2011; Nato et al., 2015), while the presence of neurogenesis in the SN still remains a matter of debate (Arzate, Guerra‐Crespob, & Covarrubiasa, 2019; Barker, Götz, & Parmar, 2018; Farzanehfar, 2018; Klaissle et al., 2012; L'Episcopo et al., 2014a; Lie et al., 2002; Xie et al., 2017).

Evidence is available that quiescent neuroprogenitors reside in the tegmental aqueduct periventricular region (Aq‐PVRs), which is close to the SNpc and harbors clonogenic NSCs endowed with DAergic potential (Hermann et al., 2006, 2009; Hermann & Storch, 2008; L'Episcopo et al., 2011a). Additionally, adult Aq‐PVR NSCs can be activated and induced to differentiate into DAergic neurons, both in vitro and after PD injury in vivo (Hermann et al., 2006, 2009; Hermann & Stork, 2008; L'Episcopo et al., 2011a, 2014a; L'Episcopo, Tirolo, Peruzzotti‐Jametti, et al., 2018a; Xie et al., 2017).

The therapeutic relevance of endogenous neurogenesis for the recovery of the injured brain and, particularly, the aged PD brain, is being actively investigated, yet remains to be elucidated (van den Barker et al., 2018; Kempermann et al., 2018; Le Grand, Gonzalez‐Cano, Pavlou, & Schwamborn, 2015; Neves et al., 2017; van den Berge et al., 2013). This avenue of research is particularly significant in light of the dramatic decline of NSC neurogenic potential in PD brain during aging and neurodegeneration, likely underlying the age‐dependent cognitive deficits and the failure to replace or repair dysfunctional or dead neurons (Anacker & Hen, 2017; Katsimpardi & Lledo, 2018; Kemperman et al., 2018; Seib & Martin‐Villalba, 2015; Takei, 2019).

The question therefore arises as to whether it is possible to counteract these age‐dependent and region‐specific restrictive mechanisms inhibiting DAergic plasticity, in order to re‐activate the endogenous neurorepair and regeneration programs in the aged PD brain. In the last decade, we have explored the functional role of adult neurogenesis in PD by addressing the molecular and cellular NSC regulatory mechanisms underlying the age‐dependent decline of neurogenic potential in a 1‐methyl‐4‐phenyl‐1,2,3,6‐tetrahydropyridine/1‐methyl‐4‐phenylpyridinium (MPTP/MPP^+^)‐induced rodent model of basal ganglia injury, investigating the potential to stimulate adult neurogenesis as a means to support neuroprotective and neurorestorative therapies (L'Episcopo, Serapide, et al., 2011b; L'Episcopo et al., 2011a, 2013, 2012, 2014a; L'Episcopo, Tirolo, Serapide, et al., 2018a, 2018b; Marchetti, 2018; Marchetti et al., 2013; Marchetti & Pluchino, 2013). Particularly, we concentrated on the key pathway regulating DAergic neurogenesis, from neurodevelopment through aging and neurodegeneration: the *wingless‐type mouse mammary tumor virus integration site (Wnt)/β‐catenin* (WβC) signalling cascade (Brodski, Blaess, Partanen, & Prakash, 2019; Inestrosa & Arenas, 2010; Maiese, 2015; Maiese, Faqi, Chong, & Shang, 2008; Marchetti, 2018; Nusse & Clevers, 2017; Nusse & Varmus, 1982; Palomer et al., 2019; Salinas, 2012; Tapia‐Rojas & Inestrosa, 2018; Toledo et al., 2017; Wurst & Prakash, 2014). The WβC‐signalling pathway is of utmost importance owing to its ability to promote tissue repair and regeneration of stem cell activity in diverse organs, and in light of its crucial role in age‐related pathogenesis and therapy of disease (Banerjee, Jothimani, Prasad, Marotta, & Pathak, 2019; Garcìa, Udeh, Kalahasty, & Hackam, 2018; Garcìa‐Velasquez & Arias, 2017; Nusse & Clevers, 2017; Tauc & Jasper, 2019; Toledo et al., 2019). The hallmark of the WβC‐pathway is the activation of the transcriptional activity of β‐catenin, the pivotal mediator of the so‐called “*canonical*” Wnt signalling. In this system, Wnt's binding to its cell surface receptors triggers a complex cascade of molecular events leading to the cytoplasmic accumulation of β‐catenin, which enters the nucleus, and associates with T‐cell factor/lymphoid enhancer binding factor (TCF/LEF) transcription factors, in turn promoting the transcription of Wnt target genes involved in a diversity of NSC functions, including survival, proliferation, and differentiation (Adachi et al., 2007; Brodski et al., 2019; Kalani et al., 2008; Piccin & Morshead, 2011). WβC‐signalling positively regulates adult neurogenesis in both the SVZ and SGZ at multiple levels: from activation of stem cells to neuronal differentiation (as reviewed by Hirota et al., 2016; Ortiz‐Matamoros et al., 2013; Varela‐Nallar & Inestrosa, 2013).

Wnts and the components of WβC‐signalling are not only widely expressed in the adult SVZ and ventral midbrain (VM), but most importantly they respond to MPTP injury and are required to trigger neurorepair programs in MPTP‐induced PD thanks to a “Wnt crosstalk dialogue” with glial cells (L'Episcopo et al., 2011b, 2011a, 2012, 2013; Marchetti et al., 2013). The critical role of astrocyte‐derived Wnt1 and WβC‐signalling was further shown in the Aq‐PVR DAergic niche, where WβC controls the fate specification of adult DAergic precursors (L'Episcopo et al., 2014a, 2014b). Conversely, aging‐dependent oxidative stress and inflammatory pathways correlate with a downregulation of WβC *‐*signalling in NSC niches and a dramatic up‐regulation of endogenous Wnt‐antagonists, with implications for SVZ neurogenesis, Aq‐PVR‐NSC activation, and DAergic self‐repair ability (L'Episcopo et al., 2012, 2013, 2014a; L'Episcopo, Tirolo, Serapide, et al., 2018a, 2018b; Marchetti et al., 2013). In 2013, these findings inspired the perspective, “*Wnt your brain be inflamed? Yes, it Wnt!*” (Marchetti & Pluchino, 2013), summarizing the potential role of an inherent self‐protective “*Wnt‐glial*” connection in the context of major NDs. Strikingly, the WβC‐pathway plays a critical role during development, in adult and aging SVZ‐, Aq‐PVR‐ and SGZ‐niches, thus providing a robust homeostatic regulatory mechanism for NSC survival, proliferation, differentiation and integration, and bearing the potential to respond to injury and regeneration with potential consequences for both non‐motor‐ and motor‐related features of PD.

Owing to the potential associations between the Wnt pathway and mitochondrial dynamics, apoptosis, and the cell cycle, which in turn affect NSC self‐renewal and differentiation (Beckervordersandforth, 2017; Beckervordersandforth et al., 2017; Chandle et al., 2016; Chong, Shang, Hou, & Maiese, 2010; Rasmussen et al., 2018; Richetin et al., 2017; Singh, Mishra, Bharti, et al., 2018b; Walter et al., 2019), we herein highlight the role of WβC*‐*signalling and its crosstalk with astrocyte‐ and microglial‐derived oxidative and inflammatory pathways in the regulation of adult neurogenesis in PD. Particular attention is paid to the exacerbated inflammation and oxidative stress associated with the upregulation of endogenous Wnt‐inhibitors.

Summarizing our work within this context, we propose a dual‐hit hypothesis governing NSC downmodulation and failure to repair. The mechanisms underlying these phenomena constitute a synergy between (a) the upregulation of proinflammatory glial pathways, (b) the decline of anti‐oxidant self‐defence mechanisms, such as the *nuclear factor erythroid‐2‐related factor 2* (Nrf2)*‐heme oxygenase 1* (Hmox1) axis, a key mediator of cellular adaptive response, and (c) the decline of astrocyte‐derived Wnts leading to NSC neurogenic impairment, with a consequent failure to recover from a PD insult. As a result, both pharmacological and cellular therapies involving the up‐regulation of WβC‐signalling and immunomodulation were reported to ameliorate the aged microenvironment, thereby promoting endogenous neurogenesis, ultimately boosting a full neurorestoration program in the aged PD brain (L'Episcopo et al., 2011c, 2012, 2013; L'Episcopo et al., 2014a; L'Episcopo, Tirolo, Serapide, et al., 2018a, 2018b; Marchetti, 2018; Marchetti et al., 2013; Marchetti & Pluchino, 2013). While little is known on WβC*‐*signalling in the PD‐injured hippocampus, it seems plausible that a comparable dysfunction of the WβC*‐*pathway may well be at play in the SGZ of the DG, with potential consequences for hippocampal neurogenesis in PD and its involvement in non‐motor symptoms of PD.

Corroborating our earlier findings, a number of recent studies have highlighted the critical importance of WβC crosstalk with survival and inflammatory pathways in inciting neurogenesis and neurorepair (Chen et al., 2018; Kalamakis et al., 2019; Kase, Otsu, Shimazaki, & Okano, 2019; Mishra et al., 2019; Morrow & Moore, 2019; Ray et al., 2018; Singh, Mishra, Bharti, et al., 2018b; Singh, Mishra, Mohanbhai, et al., 2018a; Singh, Mishra, & Shukla, 2016).

Herein, after a description of the key Wnt‐signalling components and a synopsis of adult neurogenesis in PD, we will focus on the role of WβC‐signalling as a common final pathway in mediating NSC regulation, from development to aging and PD degeneration. We aim to survey recent literature in the field supporting the upregulation of WβC as a means to re‐activate neurogenesis and incite regeneration in the injured brain, particularly in the context of modalities through which the inherent self‐repair capacities of the aged PD brain, can be engaged (Chen et al., 2018; Kase et al., 2019; Kaur, Saunders, & Tolwinski, 2017; Mishra et al., 2019; Zeng et al., 2019; Zhao, et al., 2018; Zhang et al., 2018a, 2018b, 2018ca,b,c; and following sections).

## 2 ACTIVATORS AND INHIBITORS OF THE “*WNT‐SIGNALOSOME*” AND THEIR IMPACT ON ADULT NEUROGENESIS

Wnt signalling is transduced via three different pathways, the so‐called “canonical” WβC pathway, and the “non‐canonical” Wnt/Ca^2+^ and Wnt/planar cell polarity (PCP) pathways. Among them, the WβC signalling pathway has received particular attention due to its crucial roles in regulating cell fate, proliferation and survival, whereas Wnt/Ca^2+^ and Wnt/PCP signalling are more associated with differentiation, cell polarity and migration (Nusse & Clevers, 2017). Recent studies indicate that two major branches of the Wnt signalling pathway, the WβC and Wnt/PCP pathways, play essential roles in various steps of adult SVZ and SGZ neurogenesis (as reviewed by Varela‐Nallar & Inestrosa, 2013; and Hirota et al., 2016).

**Figure 2:**
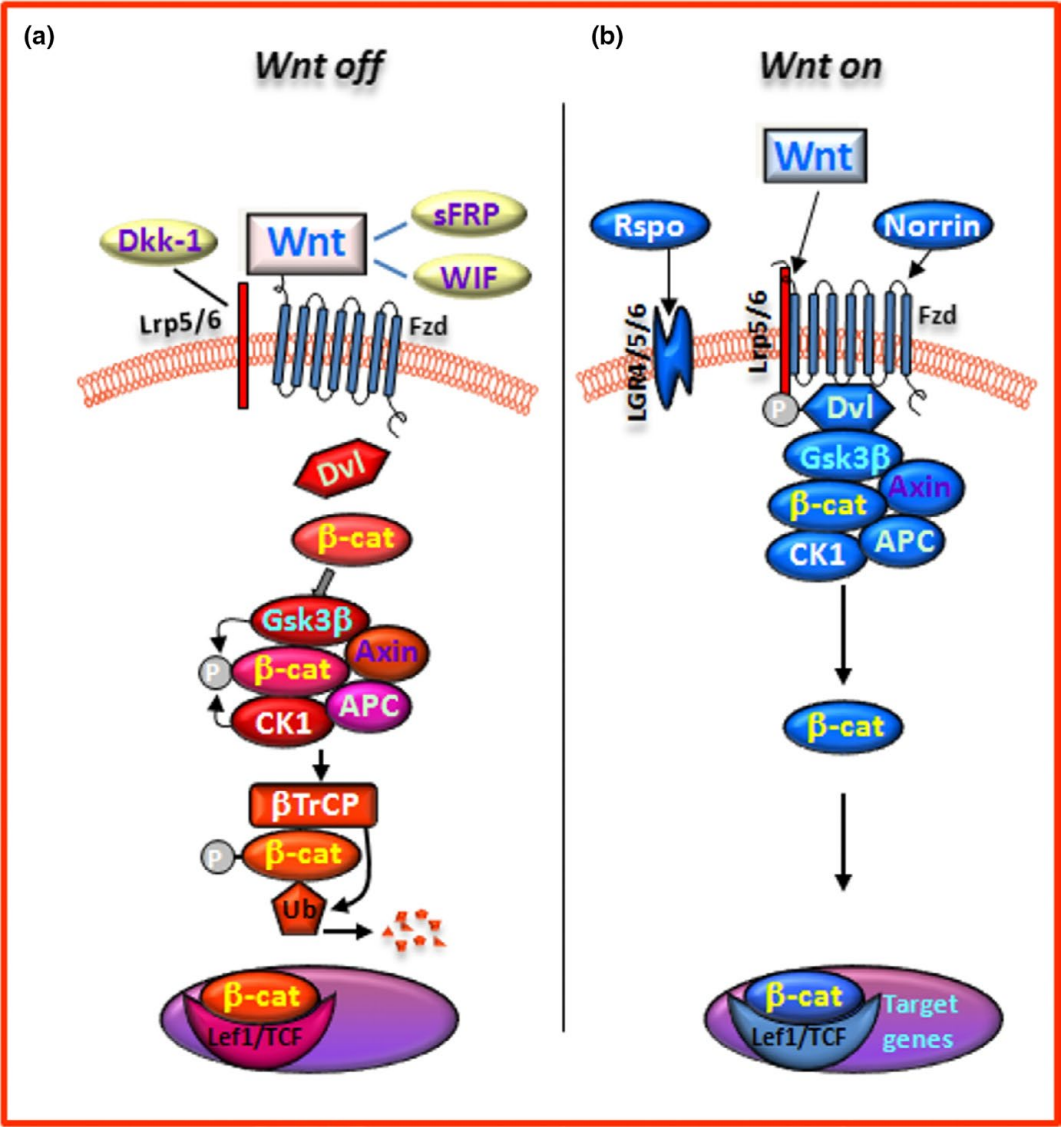
The canonical Wnt/β‐catenin(WβC) signalling pathway. In WβC pathway, Wnt signal activation is tightly controlled by a dynamic signalling complex, constituted by class Frizzled (Fzd) of the G‐protein‐coupled receptor (GPCRs) superfamily, the LDL receptor‐related protein (LRP) 5/6 coreceptors and Dishevelled (Dvl) and Axin adapters. (a) In the absence of a Wnt ligand, (*Wnt‐off*) the signalling cascade is inhibited. Cytoplasmic β‐catenin is phosphorylated and degraded via proteasome mediated destruction, which is controlled by the “destruction complex”, consisting of glycogen synthase kinase 3β (GSK3β), casein kinase 1α (CK1α), the scaffold protein AXIN, and the tumor suppressor adenomatous polyposis coli (APC) (Janda et al., 2012). As a result, the translocation into nucleus is inhibited. Interruption of WβC‐signalling also occurs in the presence of the Dkk' and secreted FZD‐related proteins (sFRPs) families of Wnt‐antagonists, or Wnt inhibitory protein, WIF. (b) Conversely, Wnt ligand binding to Fzd receptors at the surface of target cells (*Wnt‐on*) triggers a chain of events aimed at disrupting the degradation complex via Dvl phosphorylation. Then β‐catenin is separated from the destruction complex, resulting in its accumulation and stabilization in the cytoplasm (Janda et al., 2012). Subsequently, β‐catenin is imported into the nucleus where it can interact with the TCF/LEF family of transcription factors and recruit transcriptional co‐activators, p300 and/or CBP (CREB‐binding protein), as well as other components to transcribe a panel of downstream target genes. The amplification of canonical WβC‐signalling can be achieved through the participation of another set of receptors, the leucine‐rich repeat‐containing G‐protein coupled receptors (LGR4, 5, 6) and their ligands, the R‐Spondins (Rspos) and the atypical FZD4/LRP5 agonist, Norrin

In the WβC pathway, Wnt signal activation is tightly controlled by a dynamic signalling complex, called a “signalosome”, comprised of core receptors from the Frizzled (Fzd, 1–10) family of G‐protein‐coupled receptors, the low‐density lipoprotein receptor‐related protein (LRP) 5/6 co‐receptors, and the Dishevelled (Dvl) and Axin adapters (Driehuis & Clevers, 2017; Gammons, Renko, Johnson, Rutherford, & Bienz, 2016; Janda, Waghray, Levin, Thomas, & Garcia, 2012; Nusse & Clevers, 2017) (Figure 2). Non‐canonical Wnt signalling can be initiated by Wnt interactions with Fzds, or receptor tyrosine kinases such as RYK (receptor‐like tyrosine kinase) and ROR (receptor tyrosine kinase‐like orphan receptor), and regulates small GTPases [such as the Ras homolog gene (Rho), Ras‐related C3 botulinum toxin substrate (Rac), and cell division cycle 42 (CDC42) families] in a Dvl‐dependent manner (Ho et al., 2012). Non‐canonical Wnt signalling can also activate calcium flux and kinase cascades, including protein kinase C (PKC), calcium/calmodulin‐dependent protein kinase II (CaMKII) and c‐Jun N‐terminal kinase (JNK), leading to the activation of activator protein (AP)‐1‐ and nuclear factor of activated T cells (NFAT)‐regulated gene expression (Driehuis & Clevers, 2017; Gammons et al., 2016; Logan & Nusse, 2004).

Amplification of canonical Wnt signalling can be achieved through the participation of another set of receptors, the leucine‐rich repeat‐containing G‐protein coupled receptors (LGR, 4–6) and their ligands, the R‐spondins (Rspos) (Carmon, Gong, Lin, Thomas, & Liu, 2011; de Lau, Peng, Gros, & Clevers, 2014; Raslan & Yoon, 2019) (Figure 2). LGR‐Rspo complexes at the cell membrane decrease the endocytic turnover of Fzd‐LRP5/6 by neutralising the ubiquitin ligases ring finger protein 43 (RNF43) and zinc and ring finger 3 (ZNRF3) (Hao et al., 2012). The crosstalk between the canonical and non‐canonical pathways is responsible for the coordination and final outcome of Wnt signalling. Several components of the canonical Wnt pathway (Wnt‐agonists, Wnt‐receptors, and Wnt‐inhibitors) have been described in the neurogenic niches of adult mice (see Hirota et al., 2016 for a comprehensive review). Reportedly, a dynamic interplay between endogenous Wnt‐agonists and Wnt‐antagonists is at play in finely tuning the strength of Wnt signalling (Niehrs, 2012). Traditionally, Wnt‐agonists referred to as the canonical *Wnt1‐like* (including Wnt1‐3a, Wnt8, and Wnt8a) and non‐canonical *Wnt5a‐like* (including Wnt4‐7a and Wnt11) classes act as intercellular growth signals. With the exception of Norrin, an atypical Fzd4/LRP5 agonist, all 19 human Wnts share a highly conserved two‐domain structure which enables it to attach to the Fzd receptor cysteine rich domain (CRD) and bind to LRP5/6 (Janda et al., 2012).

Essentially, Wnt ligands are secreted lipid‐modified glycoproteins that act as short‐range modulators to activate receptor‐mediated signalling pathways. The lipid components of Wnts are required for protein secretion and efficient signalling (Nusse & Clevers, 2017). Wnt palmitoylation is essential for Wnt signalling and is carried out by Porcupine, an endoplasmic reticulum ‐localized O‐acyltransferase (Herr & Basler, 2012; Torres et al., 2019). Additionally, due to their hydrophobic nature, Wnts require extracellular carriers, such as the Wnt‐binding proteins Wntless and Secreted wingless‐interacting molecule (Swim), that enable secretion of the active Wnt complex by binding to lipidated Wnt (Bänziger et al., 2006).

The chief role of Wnts during DAergic neuron development is underscored by the specific requirement of a Wnt1‐induced genetic cascade for the establishment of progenitor cells and DAergic terminal differentiation in the later stages of embryogenesis (see Arenas, 2014; Brodski et al., 2019; Joksimovic & Awatramani, 2014; Prakash & Wurst, 2006; Prakash & Wurst, 2014; Zhang et al., 2015). Hence, canonical Wnt signalling is critical for midbrain DAergic progenitor specification, proliferation, and neurogenesis. The involvement of Wnts in regulating NSC activity has been established through the use of Wnt mutant mice whereby loss of Wnt1 resulted in malformation of most of the midbrain and some rostral metencephalon (see Arenas, 2014; Joksimovic & Awatramani, 2014; Prakash & Wurst, 2014). The removal of β‐catenin in tyrosine hydroxylase‐positive (TH^+^) neural progenitor cells in the VM region negatively regulates midbrain DAergic neurogenesis. Here, β‐catenin depletion interferes with the ability of committed progenitors to become DAergic neurons, resulting in adult animals with a significant loss of TH^+^ neurons in the adult VM (Tang et al., 2009). Excessive Wnt signalling is also detrimental for DAergic neuron production, adding to the general notion that morphogen dosage must be tightly regulated (Rawal et al., 2009).

Also, a large number of studies have shown a crucial participation of the WβC‐pathway at early stages of hippocampal development. Hence, the expression pattern of the *LEF1* gene of the TCF/LEF family of transcription factors, as well as other TCF/LEF proteins, are critical for the regulation of DG granule cell generation and the entire hippocampal maturation, whereas the conditional inactivation of β‐catenin in mice results in an impairment of hippocampus development (Galceran, Miyashita‐Lin, Devaney, Rubenstein, & Grosschedl, 2000; Lee, Tole, Grove, & McMahon, 2000; reviewed by Ortiz‐Matamoros et al., 2013, and Varela‐Nallar & Inestrosa, 2013). Remarkably, studies in cultured hippocampal neurons have found that β‐catenin regulates dendritic morphogenesis since the overexpression of a stabilized form of β‐catenin leads to the development of a more complex dendritic arborization (Ciani & Salinas, 2005; Salinas, 2005a,2005b; Salinas, 2012).

Activation of Wnt signalling plays a role in producing regionally homogeneous populations of NSCs and neurons. For instance, pivotal genes whose mutations are linked to PD negatively impact on WβC‐signalling (Berwick and Harvey, 2014, Berwick et al., 2017), resulting in an inhibition of the ability of human induced pluripotent cells (iPSCs) to differentiate into DAergic neurons (Awad et al., 2017; Momcilovic et al., 2014; Moya et al., 2014). Specifically, downregulation of WβC‐signalling in iPSC‐derived NSCs due to a *GBA1* mutation resulted in a dramatic decrease in the survival of DAergic progenitors (Awad et al., 2017) . Concomitanly, the positive role of pharmacological activation of WβC was demonstrated through restoration of DAergic developmental potential upon treatment with the Wnt activator CHIR99021 (Awad et al., 2017).

Both at the SVZ and SGZ niches, β‐catenin is tightly regulated via phosphorylation by the 'destruction complex', consisting of glycogen synthase kinase 3β (GSK‐3β), casein kinase 1α (CK1α), the scaffold protein Axin‐1, and the tumour suppressor adenomatous polyposis coli (APC) (Janda et al., 2012) (Figure 2). In the absence of a Wnt ligand, the signalling cascade is inhibited. Cytoplasmic β‐catenin is phosphorylated and kept at low levels via proteasome‐mediated destruction, which is controlled by the destruction complex. As a result, translocation into the nucleus is inhibited. Conversely, binding of Wnt ligands to receptors at the surface of target cells triggers a chain of events resulting in disruption of the destruction complex via Dvl phosphorylation. β‐catenin is then separated from the destruction complex, resulting in its accumulation and stabilization in the cytoplasm (Janda et al., 2012). Subsequently, β‐catenin is imported into the nucleus where it can interact with the TCF/LEF family of transcription factors and recruit transcriptional co‐activators, p300 and/or CREB‐binding protein (CBP), as well as other components to transcribe a panel of downstream target genes (Figure 2).

The enzyme GSK‐3β is a crucial inhibitor of canonical Wnt‐signalling as it leads to the degradation of β‐catenin. Inhibition of GSK‐3β activity by molecular compounds and various enzymes is an important step in the activation of the canonical Wnt signalling cascade and the downstream gene expression (Figure 2). The critical role of GSK‐3β inhibition leading to β‐catenin stabilization in VM precursors, with consequent increased differentiation into DAergic neurons, was highlighted by early studies by Arenas and collaborators (Castelo‐Branco, Rawal, & Arenas, 2004; Castelo‐Branco et al., 2006; reviewed by Arenas, 2014 and Toledo et al., 2017). Hence, two chemical inhibitors of GSK‐3β, indirubin‐3‐monoxime and kenpaullone, were found to increase neuronal differentiation in VM precursor cultures. Additionally, kenpaullone was found to increase the size of the DAergic neuron population through conversion of precursors expressing the orphan nuclear receptor‐related factor 1 (Nurr1/Nr4A2) into TH^+^ neurons, thereby mimicking the effect of canonical Wnts (Castelo‐Branco et al., 2004). These early studies documented a three‐ to five‐fold increase in precursor differentiation into DAergic neurons, paving the way for the use of GSK‐3β inhibitors to improve stem/precursor cell therapy approaches in Parkinson's disease (Arenas, 2014; Brodski et al., 2019; Esfandiari et al., 2012; Kirkeby et al., 2012; Kirkeby, Parmar, & Barker, 2017; Kriks et al., 2011; Parish et al., 2008; Parish & Thompson, 2014; Toledo et al., 2017).

While GSK‐3β antagonism, leading to β‐catenin stabilization and WβC‐signalling activation, increases NSC proliferation and neuronal differentiation, GSK‐3β overexpression inhibits neurogenesis within the SVZ, Aq‐PVR and SGZ neurogenic niches, *both *in vivo and in vitro, and promotes neuronal death (Adachi et al., 2007; Azim, Rivera, Raineteau, & Butt, 2014; Hirota et al., 2016; Kalani et al., 2008; L'Episcopo et al., 2012, 2013, 2014b, 2016; Sirerol‐Piquer et al., 2011; Varela‐Nallar & Inestrosa, 2013; Wexler et al., 2009). Stimulation of NSC proliferation can also be achieved by Wnt‐7a up‐regulation, as a result of orphan nuclear receptor Tailless (TLX) activation promoting WβC‐signalling (Qu et al., 2010).

Owing to its critical role in the regulation of a multiplicity of cellular functions, Wnt‐signalling must be kept under a strict control via a panel of endogenous Wnt‐antagonists, including proteins of the Dickkopf (Dkk) and the Sclerostin families (Cruciat & Niehrs, 2013). These molecules antagonize Wnt‐signalling by binding LRP5/6, possibly disrupting Wnt‐induced Fzd‐LRP6 dimerization (Cruciat & Niehrs, 2013). Wnt‐interfering molecules also include the secreted Fzd‐related proteins (sFRPs) and Wnt inhibitory factor (WIF) proteins, both able to bind to Wnts directly (Figure 2). Dkk1 is a potent inhibitor of SVZ‐ and SGZ‐neurogenesis in vivo*, *ex vivo*,* and when modelled in vitro (Lie et al., 2005; Varela‐Nallar & Inestrosa, 2013; Hirota et al., 2016; [see also next sections], while the β‐catenin binding homeodomain‐interacting protein kinase‐1 (HIPK1) is sharply up‐regulated in the postnatal stage, modulating β‐catenin activity (Marinaro et al., 2012). Conversely, decreased wild‐type p53‐induced phosphatase 1 (WIP1) decreased expression leads to increased Dkk3‐dependent inhibition of WβC‐signalling in the SVZ, resulting in age‐dependent declines in neurogenesis and olfactory function in mice (see Qiu et al., 2018; Zhu et al., 2014). Hence, permanent middle cerebral artery occlusion in *WIP1*‐knockout mice was found to result in inhibited neurological functional recovery, reduced expression of doublecortin (DCX), and inactivation the WβC‐signalling pathway, whereas pharmacological activation of WβC‐signalling compensated for this *WIP1* knockout‐induced deficit in neuroblast formation (Qiu et al., 2018).

Adding a further level of complexity, miRNAs (short noncoding RNAs) are increasingly emerging as critical regulators of Wnt‐signalling (Song et al., 2015) and, vice versa, Wnt‐signalling components can modulate miRNA activity. In a key finding, Anderegg and colleagues (2013) uncovered a regulatory circuit between LIM homeobox transcription factor 1‐beta (LMX1B) and miR‐135a2 that modulates Wnt1/Wnt signalling which in turn determines the size of the midbrain DAergic progenitor pool. On the basis of bioinformatics and luciferase assay data, the authors suggested that miR‐135a2 modulates LMX1B and many genes in the Wnt signalling pathway (Anderegg & Awatramani, 2015; Anderegg et al., 2013). Chmielarz et al., (2017) underscored the crucial role of Dicer, an endoribonuclease essential for miRNA biogenesis and other RNAi‐related processes, for maintenance of adult DAergic neurons; a reduction of Dicer in the VM and altered miR expression profiles were observed in laser‐microdissected DAergic neurons of aged mice (Chmielarz et al., 2017).

miRNAs are involved in stem cell fate and self‐renewal and regulate the expression of stem cell genes. In 2016, Rolando et al. reported that deletion of the miRNA‐processing ribonuclease Drosha in the adult DG activates oligodendrogenesis and reduces neurogenesis at the expense of gliogenesis. Pons‐Espinal et al. (2017) addressed the synergic functions of miRNAs in determining the neuronal fate of adult NSCs. Particularly, Dicer is required for the generation of new neurons, but not astrocytes, in the adult murine hippocampus (Pons‐Espinal et al., 2017). Specifically, β‐catenin controls Dicer gene expression, thus highlighting WβC‐Dicer crosstalk as an important step in determining neuronal fate. Also, emerging evidence implicates several miRNAs in controlling WβC; for example, overexpression of miR‐21 in primary rat NSCs in vitro was shown to promote NSC proliferation and neural differentiation via the WβC signalling pathway (Zhang, Zhang, Deng, et al., 2018a; Zhang, Shi, et al., 2018b; Zhang, Zhang, Yang, et al., 2018c). Thus, both miRNAs and Wnt‐signalling pathways form a network (Ashmawy et al., 2017) that is likely to play a significant role in adult neurogenesis.

Taken altogether, a picture emerges of the complexity of the Wnt signalling cascade whereby the outcome of WβC‐signalling activation is context‐dependent, with β‐catenin activating different and sometimes opposing genetic programs depending on tissue/cellular specificity, the availability of receptor/co‐receptors and signalling partners, pathological conditions, and the age of the host. Due to the vital action of this signalling pathway in development and systems maintenance, its dysregulation may culminate in a broad range of diseases, including neurodegeneration and cancer (Nusse & Clevers, 2017).

Thus, WβC‐signalling is a likely prominent actor in tipping the balance of adult PD brain NSCs due to a concerted action of diverse upstream and downstream modulatory signals impacting within the specialized neurogenic niches.

## 3 WNT/β‐CATENIN SIGNALLING IN PD

### 3.1 A dynamic interplay of positive and negative regulators coordinates adult SVZ and SGZ neurogenesis in PD

**Figure 3:**
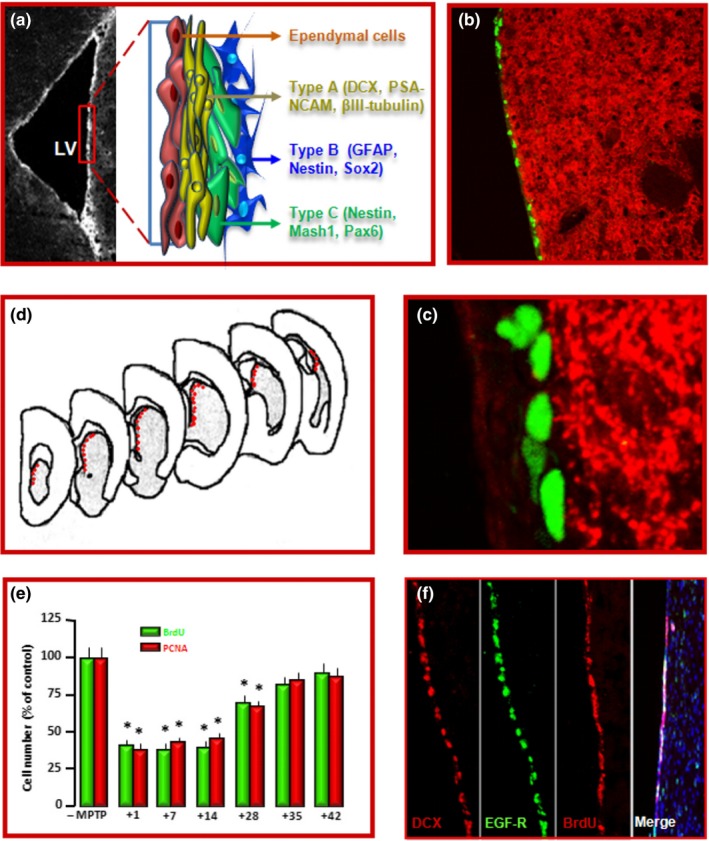
Location, proliferation and dopaminergic innervation of SVZ‐NSCs. (a) Microscopic brain image at the level of the striatal SVZ. In the inset, a schematic drawing of the four SVZ‐cell types: 1. slowly dividing SVZ astrocytes (type B cells), 2. rapidly dividing transit‐amplifying cells (type C cells, TAPs), 3. migrating neuroblasts (type A cells), and 4. ependymal cells (type E cells) (Doetsch et al., 1997, 1999). (b, c) Nigrostriatal DAergic neurons originating in the SN project to the SVZ. Dual immunofluorescent staining with dopamine transporter (DAT, in red) and bromodeoxiuridine (BrdU, green) showing a dense network of DAT expressing neurons innervating the SVZ (b; and higher magnification in c). (d–f) schematic representation of cell counting performed in coronal sections through the SVZ (d); stereological estimations of BrdU and proliferating cell nuclear antigen (PCNA)‐positive cells (e), and representative stainings of DCX^+^, EGF‐R^+^, BrdU^+^, counterstained with the nuclear marker 4,6‐diamidino‐2‐phenylindole, Dapi (blue) (f), indicating that MPTP‐induced basal ganglia injury resulted in a biphasic time‐dependent response: a down‐regulation of SVZ‐NSC proliferation followed by a return to pre‐MPTP levels

As adult neurogenesis occurring at the SVZ and SGZ levels is a vital ongoing process in the adult brain, its disruption may contribute to various disturbances including reduced neuronal plasticity, deficits in olfaction, cognitive dysfunction, and/or mental health disorders, that may precede and/or accompany PD onset and progression. Therefore, the tight regulation of the sequential steps of adult neurogenesis should be finely orchestrated by a wide panel of transcription factors and epigenetic mechanisms coordinating the progression of neurogenesis (Bond et al., 2015; Hsieh, 2012; Ming & Song, 2005). At the SVZ, located along the ependymal cell layer of the lateral ventricles (Figure 3), four major cell types dynamically interact with each other: slowly dividing SVZ‐glial fibrillary acidic protein (GFAP)‐positive astrocytes (type B cells), rapidly dividing TAPs (type C cells), migrating neuroblasts (type A cells), and ependymal cells (type E cells) (Doetsch, Caillé, Lim, García‐Verdugo, & Alvarez‐Buylla, 1999; Doetsch, García‐Verdugo, & Alvarez‐Buylla, 1997) (Figure 3). Type B cells exhibit a radial morphology and extend a basal process to terminate on blood vessels and an apical process with a primary cilium contacting the cerebrospinal fluid (CSF) in the ventricle (Mirzadeh et al., 2008). B cells give rise to TAPs (Doetsch et al., 1999, 1997), which rapidly divide to become neuroblasts (A cells). Neuroblasts then form a chain and migrate following the rostral migratory stream (RMS) to the olfactory bulb, OB, where they become granular and periglomerular interneurons involved in odor discrimination (Lledo & Valley, 2016) (Figures 1 and 3). In the adult mouse DG, radial glia‐like precursors within the SGZ serve as one type of quiescent NSCs and continuously give rise to both dentate granule neurons and astrocytes (Bonaguidi et al., 2011; Figure 1). Here, different populations of progenitors with different properties have been described, enhancing the complexity of the cellular and molecular mechanisms underlying regulation of adult neurogenesis (Bonaguidi et al., 2011; Encinas et al., 2011; Ming & Song, 2005). Reportedly, the local neurogenic niche both harbours NSCs and regulates their development. Within the SVZ “stem cell niche” (Alvarez‐Buylla et al., 2001; Alvarez‐Buylla & Lim, 2004; Fuentealba et al., 2012; Lim & Alvarez‐Buylla, 2016; Song et al., 2002), DAergic innervation collaborates with a wide variety of factors including growth and neurotrophic factors, morphogens, cytokines, CSF‐derived regulatory molecules and key components of the WβC‐signalling pathway, contributing to SVZ regulation (Adachi et al., 2007; Borta & Holinger, 2007; Kalani et al., 2008; L'Episcopo et al., 2012, 2013; O'Keeffe Barker, & Caldwell, 2009; O'Keeffe, Tyers, et al., 2009b; Pluchino et al., 2008; Silva‐Vargas et al., 2016). Thus, NSCs can sense both central and systemic changes via circulating factors and are targeted by multiple CSF signals (Silva‐Vargas et al., 2016). Among the niche components of the hippocampal SGZ, which include blood vessels, ependymal cells, and mature neurons (Ming & Song, 2005), hippocampal astrocytes have a key role as they instruct the neuronal fate of cultured adult neural progenitors via WβC‐signalling (Lie et al., 2005; Song et al., 2002; Varela‐Nallar & Inestrosa, 2013). Accordingly, lentivirus‐mediated blockade of Wnt signalling reduces the number of immature new neurons in the adult dentate gyrus and impairs hippocampus‐dependent spatial‐ and object‐recognition memory (Jessberger et al., 2009).

Not surprisingly, different physio‐pathological conditions and pharmacological stimuli dynamically modulate both SVZ‐ and SGZ‐NSCs. Among the positive factors, DAergic innervation and DA‐agonists, serotonin, exercise, enriched environment, learning, estrogens, and antidepressant drugs have been variously documented to stimulate NSC neurogenic potential (Baker, Baker, & Hagg, 2004; Hoglinger et al., 2004; Chiu et al., 2015; Ehninger et al., 2011; Kempermann, Kuhn, & Gage, 1997; Kempermann, Kuhn, & Gage, 1998; Kodali et al., 2016; Kohl et al., 2016; van Praag et al., 2005; Salvi et al., 2016; Winner et al., 2009a, 2011a,b). Conversely, DAergic neurotoxins, aging, inflammation, stress and chronic exposure to certain toxins or drugs decrease the generation of new neurons (Chandel et al., 2016; Das, Gangwal, Damre, Sangamwar, & Sharma, 2014; Hain et al., 2018; Iwata et al., 2010; Klein et al., 2016; L'Episcopo et al., 2011c, 2012, 2013; Marchetti & Pluchino, 2013; van Praag et al., 2005; Seib & Martin‐Villalba, 2015; Sung, 2015; Veena et al., 2011; Villeda et al., 2011). NSCs are extremely vulnerable to a wide range of insults and toxic exposures, such as the neurotoxicants used in experimental models of basal ganglia injury (Cintha et al., 2018; He et al., 2006; He, Uetsuka, & Nakayama, 2008; L'Episcopo et al., 2012; Shibui et al., 2009). Remarkably, exposure to the herbicide paraquat, which is associated with an increased risk of idiopathic PD, results in a pro‐inflammatory senescence‐associated secretory phenotype capable of damaging neuronal, glial and NSC cells, and therefore likely contributing to DAergic neurodegeneration (Chinta et al., 2018). Aging, especially, represents a key driver of neurogenic impairment as a result of a reduced proliferation and differentiation of NSCs both at SVZ and SGZ levels (Ahlenius, Visan, Kokaia, Lindvall, & Kokaia, 2009; Daynac, Morizur, Chicheportiche, Mouthon, & Boussin, 2016; Enwere et al., 2004; Luo, Daniels, Lennington, Notti, & Conover, 2006). NSCs still retain their capacity for proliferation and differentiation into functional neurons, despite lower numbers in the aged brain (Ahlenius et al., 2009). Recent evidence also suggests that mitochondrial dysfunction represents an important cause of the age‐related decline in neurogenesis, as exposure of the SGZ or SVZ to mitogens or enhancement of mitochondrial function restored neurogenesis (Beckervordersandforth et al., 2017). Notably, the mitochondrial transcription factor A (Tfam), a mitochondrial DNA (mtDNA)‐binding protein essential for genome maintenance, has recently gained attention, as its putative dysfunction may play an important role in neurogenesis defects in the aging hippocampus (Beckervordersandforth et al., 2017) and aging‐dependent neurodegeneration (Kang, Chu, & Kaufman, 2018). Tfam has been shown to play a central role in the mtDNA stress‐mediated inflammatory response and recent evidence indicates that decreased mtDNA copy number is associated with several aging‐related pathologies (Kang et al., 2018). Of special mention, besides its crucial role for the development, maintenance and protection of midbrain DAergic neurons, the relevance of the transcription factor Nurr1 for adult hippocampal neurogenesis in PD has been recently highlighthed, and several lines of evidence indicate that pharmacological stimulation of Nurr1 can improve behavioral deficits via an increase in hippocampal neurogenesis (Kim et al., 2015; Kim et al., 2016 and section 6.4.1).

One key compartment that is dramatically affected in PD is represented by DAergic innervation of the SVZ and SGZ. Hence, nigrostriatal DAergic neurons originating in the SN project to the SVZ in mice, primates, and humans (Borta & Hoglinger, 2007; Freundlieb et al., 2006; Höglinger, Arias‐Carrión, Ipach, & Oertel, 2014; Hoglinger et al., 2004). Within the SVZ niche, DAergic terminals create a dense network of fibers innervating the SVZ (Figure 3 and Figure S1). Accordingly, DA receptors are widely expressed in the SVZ region and are actively involved in the modulation of neurogenesis (Van Kampen et al., 2004). In the hippocampal SGZ, DAergic neurites remain in close contact with NSCs and make their synaptic connections with granular cells in the DG, and it is thought that DA provides an environment for proliferation and differentiation of NSCs (Höglinger et al., 2014, 2004). Accordingly, a number of studies have shown that DA depletion in animal models of PD reduced the proliferation of NSCs in the SVZ and SGZ (recently reviewed by Winner & Winkler, 2015) . Additionally, besides DA, a dysfunction of the serotonergic system projecting to the hippocampus has been reported to impact on adult neurogenesis and suggested to contribute to early non‐motor symptoms of PD, such as anxiety and depression (Kohl et al., 2016).

NSCs have been identified in the SVZ of the aged human brain (Apple et al., 2017; Berge et al., 2011; Bergman, Spalding, & Frisén, 2015; Donega et al., 2019; Leonard et al., 2009; Radad et al., 2017; van den Berge et al., 2010; Wang et al., 2012; Winner, Vogt‐Weisenhorn, et al., 2009b). NSCs, which are GFAP (δ isoform) and nerve growth factor receptor (NGFR, a.k.a. CD271) positive, were shown to proliferate and differentiate towards neurons and glial cells in vitro, then supporting the potential ability to stimulate these NSCs to regenerate the injured brain (van den Berge et al., 2011; Donega et al., 2019). In post‐mortem PD brains a deregulation of SVZ neurogenesis was revealed, with decreased NSC proliferation correlated with the progression of PD, while L‐DOPA treatment appeared to increase NSC numbers. Also, levels of epidermal growth factor (EGF) and its receptor (EGFR) are decreased in the PD Str and the prefrontal cortex of PD patients, and EGFR^+^‐NSC numbers are decreased in the SVZ of PD patients (O'Keeffe, Barker, et al., 2009; O'Keeffe, Tyers, et al., 2009b). At the mesencephalic level in idiopathic human PD, multipotent NSCs isolated from the SN appeared to lack key factors required for neuronal differentiation as they must be co‐cultured with embryonic stem cell‐derived neural precursors to obtain neurons (Wang et al., 2012). Within the hippocampal SGZ, neurogenesis is severely impaired, which may be associated with hippocampal atrophy. Specifically, Hoglinger et al. (2004) reported a decreased number of DG cells expressing nestin and βIII‐tubulin in three PD patients and five patients suffering from PD with dementia (PDD) compared with three controls, with PDD showing a more severely decreased number of nestin‐expressing cells in the human DG. Additionally, Winner et al. (2012) showed a reduction in the number of SRY box transcription factor 2 (Sox2)‐expressing cells in the DG, in dementia with Lewy bodies, and this was paralleled by an increase in α‐syn‐positive cells (see Miller & Winkler, 2015).

Of special importance, the Str, previously defined as a non‐neurogenic region, was recently found to generate neuroblasts in response to different types of brain injury (Luzzati et al., 2011; Nato et al., 2015). Striatal neurogenesis has also been observed in adult non‐human primates such as the squirrel monkey (Bedard et al., 2002). Remarkably, Ernst et al., using a birth dating approach based on the incorporation of nuclear‐bomb‐test‐derived ^14^C in the DNA of proliferating cells, reported that new neurons are generated in the adult Str in humans (Ernst et al., 2014), thereby indicating that the possibility exists to stimulate such a mechanism to sustain DAergic striatal reinnervation in PD.

Using well recognized neurotoxic rodent PD models such as the MPTP‐ or 6‐hydroxydopamine (6‐OHDA)‐based models of basal ganglia injury, a number of studies have established that selective nigrostriatal DAergic degeneration in rodents and primate PD models markedly impairs SVZ and/or SGZ neurogenic potential (Chiu et al., 2015; Freundlieb et al., 2006; Hoglinger et al., 2004; L'Episcopo et al., 2012, 2013; Lao, Lu, & Chen, 2013; O'Keeffe, Barker, et al., 2009; O'Keeffe, Tyers, et al., 2009b; Salvi et al., 2016; van Kampen & Eckman, 2006; Van Kampen et al., 2004; Winner, Desplats, et al., 2009a; Winner et al., 2011; Winner, Vogt‐Weisenhorn, et al., 2009b; Winner & Winkler, 2015). In PD, hippocampal non‐motor functions such as spatial learning and memory are impaired, and in several studies the impaired neurogenesis following DA depletion correlated with certain cognitive deficits observed in PD (Das et al., 2014; Klein et al., 2016; Lesemann et al., 2012; Sung, 2015). Other studies, however, showed no difference in proliferation or differentiation of newborn cells in the SGZ of the DG after DAergic lesions (Ermine et al., 2018 and refs therein). As different factors including age, sex, inflammation, the brain region examined, and the post‐injury interval considered, significantly affect the NSC response to injury (Figure 3; and Figure S1) (Khan, Wakade, de Sevilla, Brann, 2015; L'Episcopo, 2013, 2012; L'Episcopo, Tirolo, Serapide, et al., 2018b; Tatar, Bessert, Tse, Skoff, 2013, and following sections), it seems plausible that impairment of SVZ and SGZ neurogenesis in PD may well depend on both DAergic and non‐DAergic related mechanisms.

Another critical factor impacting on adult neurogenesis is the presence of gene mutations. Looking at the overexpression of *SNCA* (either WT or mutant forms), a number of reports suggest a decrease in the number of newly generated neurons in the DG, OB and SN of the adult brain, including also the DAergic neurons in both the OB and SN (see Le Grand et al., 2015 and refs therein). Transgenic mice overexpressing the *LRRK2* G2019S mutation display a significant decrease in the number of proliferating cells in the adult DG, SVZ and RMS, and decreased neurogenesis and DA neurogenesis, as well as decreased survival of newly generated neurons in the OB (Winner, Desplats, et al., 2009a). The combined exposure to the agrichemicals maneb and paraquat or MPTP, powerfully interact with PD mutations and further decrease both SVZ‐ and SGZ‐NSC neurogenic potential, which may contribute to the overall PD pathology (Desplat et al., 2012; Le Grand et al., 2015; Peng & Andersen, 2011; Winner & Winkler, 2015). Conversely, a loss of function mutation of *PINK1* (a protein important for mitochondrial quality control via autophagic degradation of damaged mitochondria) negatively impacts on adult neurogenesis. PINK1 deficiency in mice impairs gait, olfaction and serotonergic innervation of the olfactory bulb (Glasl et al., 2012). Recently, Agnihotri and collaborators (2019), reported that PINK1 deficiency is associated with increased deficits of adult hippocampal neurogenesis and lowers the threshold for stress‐induced depression in mice.

As a whole, adult neurogenesis is highly susceptible to multiple “risk factors” for PD, including genetic mutations, DAergic innervation, aging and exposure to environmental toxins. The presence of NSCs in the human PD brain corroborates the potential for developing therapeutic strategies aimed at stimulating the endogenous NSC pool to sustain neurorepair and/or target the pre‐motor symptoms of PD. However, little is known about the molecular and cellular mechanisms of aging and PD‐induced NSC down‐modulation. Based on our own studies and the background literature, the hypothesis being highlighted here relies on the identification of WβC‐signalling as a common hallmark of both extrinsic and intrinsic signals impacting on NSC homeostatic regulation.

### 3.2 Dysfunctional Wnt/β‐catenin signalling as a critical event in MPTP‐dependent SVZ impairment: in vivo studies

Using immunohistochemistry to localize β‐catenin in the SVZ of saline‐ and MPTP‐treated mice to unravel the potential role of WβC‐signalling in SVZ‐NSCs during PD, we first identified a significant proportion of β‐catenin*^+^* cells co‐expressing bromodeoxyuridine (BrdU), indicative of proliferation (L'Episcopo et al., 2012). By contrast, MPTP treatment sharply down‐regulated the β‐catenin immunofluorescence signal and β‐catenin^+^ cell proliferation (Figure S2). Since β‐catenin is expressed in type C cells (Adachi et al., 2007) and given that MPTP reduced the proliferation of type C cells, we next addressed the relevance of the SVZ‐WβC‐signalling pathway in MPTP‐induced SVZ neurogenic impairment, by the use of WβC‐signalling activators or inhibitors. Activation of WβC was performed using a specific GSK‐3β antagonist, AR‐AO14418 (AR; Osakada et al., 2007), by either systemic injections or local SVZ administration by intracerebroventricular (icv) infusion. In mice exposed to MPTP, GSK‐3β‐antagonist administration resulted in a sharp increase in β‐catenin expression and cell proliferation in the SVZ ipsilateral to the infusion, therefore counteracting the MPTP‐induced decrease in proliferating cell nuclear antigen‐positive cells and β‐catenin expression in the SVZ (Figure S2). These findings further indicated that the acute exogenous activation of WβC‐signalling during the temporal window of maximal neurogenic impairment may overcome the disrupted neurogenesis observed in the SVZ of MPTP mice in vivo (L'Episcopo et al., 2012). Conversely, WβC antagonism in intact mice using the soluble inhibitor Dkk1, infused via icv, was able to mimic the MPTP‐induced decrease in β‐catenin^+ ^cells (L'Episcopo et al., 2012). This inhibitory effect was efficiently counteracted by concomitant activation of the downstream transcriptional effector, β‐catenin, with systemic AR injections (Figure S2). Very recently, the beneficial effect of WβC activation was reported (Singh, Mishra, Bharti, et al., 2018b; Singh, Mishra, Mohanbhai, et al., 2018a) in the 6‐OHDA rat model of PD. Here, activation of WβC‐signalling using the specific GSK‐3β inhibitor SB216763 efficiently counteracted the 6‐OHDA‐induced neurogenic impairment at both the SVZ and SGZ levels. In the hippocampus, GSK‐3β inhibition enhanced dendritic arborization and survival of granular neurons and stimulated NSC differentiation towards the neuronal phenotype in the DG (Singh et al., 2018b; Singh, Mishra, Mohanbhai, et al., 2018a).

Together, these in vivo findings suggested a disruption of WβC‐signalling associated with decreased proliferation and neuroblast formation in the MPTP‐injured SVZ, and further documented the ability of pharmacological activation of β‐catenin‐mediated signalling to counteract the impaired neurogenic potential of MPTP or Dkk1‐infused mice. Coupled to the recent findings obtained at the SGZ level, these data highlight WβC as a key pathway for the regulation of NSC homeostasis during basal ganglia injury induced by the neurotoxins MPTP and 6‐OHDA, both in the SVZ‐ and hippocampal SGZ‐niches.

### 3.3 WβC‐signalling is a key player in SVZ‐NSC homeostasis in PD mice: ex vivo and in vitro findings

Looking at Wnt signalling components using western blot analysis we found a decreased β‐catenin signal in NSCs isolated from MPTP‐ but not saline‐treated mice, whereas the active GSK‐3β signal was sharply increased. We then used quantitative real‐time PCR to show that expression levels of Axin‐2, a direct Wnt target induced by WβC activation (Jho et al., 2002), were down‐regulated in MPTP‐NSCs as compared to Axin‐2 expression levels of saline‐NSCs, thereby supporting MPTP‐induced inhibition of WβC signalling activity in SVZ cells (L'Episcopo et al., 2012).

Next, we took advantage of a small interfering RNA (siRNA) strategy (He & Shen, 2009) to further examine the relationship between the β‐catenin signalling pathway in MPTP‐induced neurogenic impairment, testing the role of β‐catenin and GSK‐3β, in NSCs isolated from MPTP‐ and saline‐treated mice. Transient transfection of NSCs from MPTP‐treated mice with *anti‐Gsk3b* siRNA resulted in a significant increase in the percentage of BrdU^+^ and microtubule‐associated protein 2a (MAP2a)^+^ cells relative to those treated with a control siRNA, suggesting that MPTP‐induced neurogenic SVZ‐impairment is at least in part mediated by GSK‐3β over‐activation (L'Episcopo et al., 2012). Reciprocally, when NSCs from saline‐treated mice were transiently transfected with anti‐β‐catenin siRNA (He & Shen, 2009), we observed a reduced percentage of BrdU^+^ and MAP2a^+^ cells, thereby corroborating the crucial role of β‐catenin in maintaining SVZ‐NSC neurogenic potential.

To look more deeply into the MPTP‐induced impairment of NSC homeostasis, we addressed the effect of a direct exposure of NSCs to MPP^+^ (the neurotoxic oxidation product of the MPTP prodrug) in vitro and found, here again, that the β‐catenin signal was downregulated in face of a sharp up‐regulation of active GSK‐3β signal. In stark contrast, exogenous activation of WβC*‐*signalling with the selective GSK‐3β antagonist AR, or the Wnt ligand Wnt1, efficiently reversed both the decreased β‐catenin signal and MPP^+^‐induced decrease in NSC proliferation and neuron differentiation (L'Episcopo al. 2012).

Together, the “in vivo*”*, “ex vivo” and “in vitro” results clearly established MPTP/MPP^+^‐induced inhibition of WβC‐signalling activity in SVZ‐NSCs as a crucial step in the neurogenic impairment of PD mice.

### 3.4 Wnt signalling crosstalk with neuroinflammatory pathways contributes to SVZ plasticity in PD

Within the mechanisms affecting adult neurogenesis in brain diseases, oxidative, and especially nitrosative stress, are likely to play critical roles, given their contribution to the aging process and the development of age‐related diseases (Shetty et al., 2018). In PD especially, glial inflammatory mechanisms have long been recognized to contribute to both nigrostriatal degeneration and self‐repair (see Gao et al., 2011; Hirsh & Hunot, 2009; L'Episcopo, Tirolo, Peruzzotti‐Jametti, et al., 2018a; L'Episcopo, Tirolo, Serapide, et al., 2018b; Marchetti & Abbracchio, 2005; Marchetti, et al., 2005a, 2005b, 2005c; McGeer & McGeer, 2008; Marchetti et al., 2011). A critical role of both central and peripheral inflammation has become evident, with a panel pro/anti‐inflammatory cytokines regulating neurogenesis (Butovsky et al., 2006; Chinta et al., 2013; Ekdhal, Kokaia, & Lindvall, 2009; Jakubs et al., 2008; L'Episcopo et al., 2011a; Monje et al., 2003; Pluchino et al., 2008; Shetty et al., 2018; Tepavcevic et al., 2011; Thored et al., 2009; Villeda et al., 2011; Vucovic et al., 2012; Wallenquist et al., 2012). In our studies (L'Episcopo et al., 2012, 2013), we investigated the inflammatory SVZ response as a function of aging and MPTP exposure, addressing the contribution of astrocytes and microglia in MPTP‐induced SVZ impairment (Figure S3). We explored the nature of the astrocyte and microglial‐derived factors involved, using astrocytes and macrophages/microglia acutely isolated ex vivo, from saline‐ or MPTP‐treated mice (L'Episcopo et al., 2012, 2013). In the same years, by using NSC culture techniques, intraventricular tumor necrosis factor (TNF)‐α infusion and the 6‐OHDA mouse model, mimicking PD‐associated neuroinflammation, Worlitzer et al. (2012) showed significant detrimental effects on SVZ‐NSCs, due to a decreased generation of DCX^+^ neuroblasts, but the signalling mechanism(s) underlying such inflammatory‐mediated detrimental effects were not fully clarified. Then, in our in vivo studies of MPTP injury, we linked early SVZ impairment to WβC down‐regulation in PD mice and the early up‐regulation of microglial phagocyte oxidase (PHOX) and inducible‐nitric oxide synthase (iNOS), generating the highly toxic peroxynitrite fingerprint 3‐nitrotyrosine and defining the exacerbated, proinflammatory, microglial M1 phenotype. Such up‐regulation of oxidative and nitrosative status likely contributed to NSC mitochondrial dysfunction, thus increasing NSC vulnerability to cell death (L'Episcopo et al., 2012, 2013). Additionally, the over‐activation of microglia, coupled with the marked astrocytosis associated with a sharp increase in major proinflammatory cytokines observed in vivo, shifted the SVZ niche to a harmful environment for neuroblast proliferation and differentiation.

The direct effects of the changing glial environment were next studied ex vivo and in vitro*,* using different glial‐NSC co‐culture paradigms, along with pharmacological antagonism/RNA silencing experiments coupled to functional studies. Hence, we provided the first evidence supporting a critical role of SVZ reactive astrocytes and macrophages/microglia in the remodeling of the SVZ niche of parkinsonian mice, identifying WβC*‐*signalling as a critical player in NSC‐glia crosstalk.

**Figure 4:**
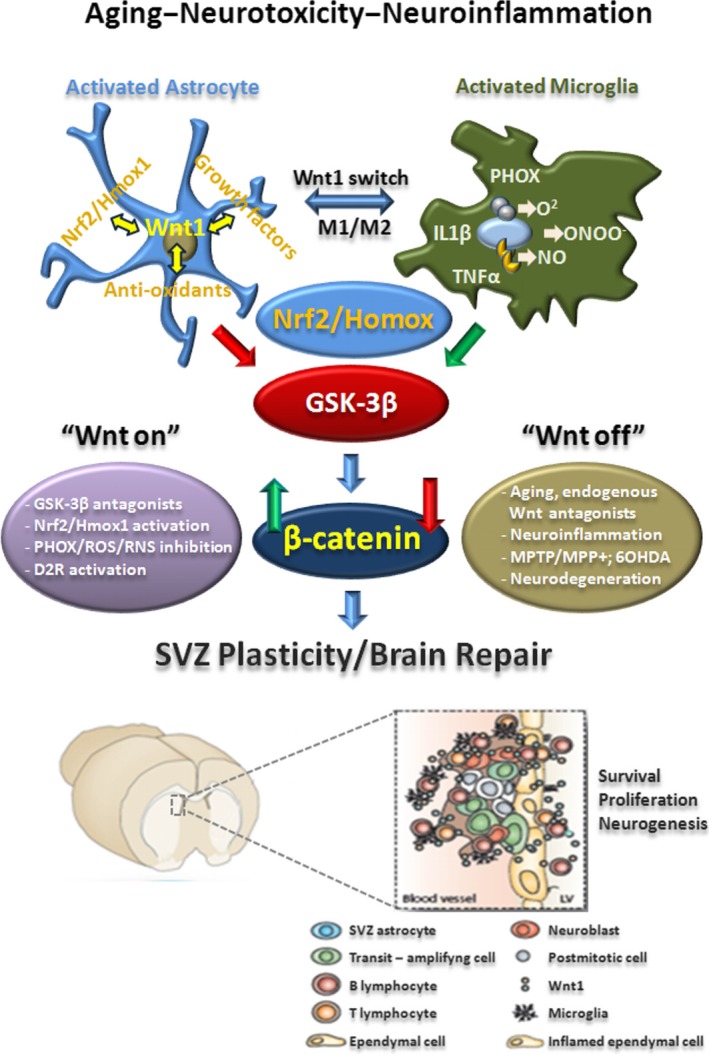
Cross talk dialogue between inflammatory and WβC‐signalling pathways in MPTP‐induced SVZ plasticity is lost with age. A simplified scheme summarizing MPTP‐induced neuroinflammation and SVZ plasticity in young and aged mice via modulation of WβC‐signalling (*“Wnt on; Wnt off”*) is shown. In young mice, during the degeneration phase, hyperactivated M1 microglia contributes to the impairment of SVZ neurogenesis at different levels. By increasing oxidative and nitrosative stress and in synergy with MPTP/MPP^+^ direct toxicity, microglial‐derived mediators (PHOX‐derived ROS, iNOS‐derived NO, and peroxynitrite) may act as molecular switch for cell signalling pathways critically involved in the physiological control of NSC homeostasis, with harmful consequences for astrocyte and NSC physiology, at least in part through GSK‐3*β* activation, followed by phosphorylation and consequent degradation of β‐catenin. In young mice, after the acute inflammatory and degenerative phase, a regulatory circuit linking microglial activation and proinflammatory cytokine to Nrf2‐ARE protective pathway in SVZ, provides an efficient self‐adaptive mechanism against inflammatory/neurotoxin‐induced oxidative stress, switching the M1 microglial harmful phenotype, thus mitigating inflammation with a return to pre‐MPTP conditions. By contrast, the aging process, in synergy with MPTP exposure, negatively impacts on astrocytic Nrf2‐driven Hmox1 response within the SVZ niche in vivo. Hence, this process, resulting from an age‐dependent dysregulation of astrocyte‐microglia interactions, contributes to the exacerbated oxidative and inflammatory SVZ status and the decline of astrocyte Wnt‐dependent regulation, finally leading to NSC neurogenic impairment and loss of SVZ plasticity The mutual role of astrocyte–microglial interactions in the plasticity of SVZ response to MPTP is exemplified by the astrocyte's ability to overcome microglial inhibitory effects, also via cross talk with *Wnt/β‐catenin* signalling. Pharmacological mitigation of inflammation and oxidative stress or GSK‐β antagonism upregulates *β‐catenin* and successfully rescues NSC proliferation and neuroblast formation, a process associated with striatal DAergic neuroprotection, with further positive modulation of SVZ proliferation via D2 receptor (D2R) activated mechanisms

Significantly, GSK‐3β/β‐catenin disruption appeared to be a potential candidate mediator of MPTP‐microglia‐induced NSC impairment. Such a hypothesis was supported by different lines of evidence, with the pharmacological inhibition of microglial reactive oxygen species (ROS) and reactive nitrogen species (RNS): (i) normalizing GSK‐3β activity; (ii) affording a significant reversal of NSC impairment; and (iii) up‐regulating β‐catenin levels in NSCs, corroborating crosstalk between inflammatory and WβC*‐*signalling components in SVZ‐NSCs (Figure 4).

## 4 THE INFLUENCE OF AGING ON WNT/β‐CATENIN SIGNALLING IN THE CONTEXT OF PD

### 4.1 Aging and MPTP‐induced exaggerated neuroinflammation: consequences for Wnt/inflammatory crosstalk

#### 4.1.1 Depletion of SVZ‐NSC starts by middle age

The process of aging is accompanied by a marked decrease in the neurogenic potential of the SVZ, as revealed by sharp decreases in the total number of BrdU^+^ cells, DCX^+^ neuroblasts, and EGFR^+^ cells (Ahlenius et al., 2009; Enwere et al., 2004; L'Episcopo et al., 2012, 2013; Luo et al., 2006). A significant reduction in BrdU^+^ cells and DCX^+^ neuroblasts has already occurred by middle‐age, and a further, albeit smaller, decline in BrdU^+^ cells and DCX^+^ neuroblasts is observed in aged mice. The type C cell compartment is particularly affected, since a sharp loss of EGFR^+^ cells is observed from middle‐age on (L'Episcopo et al., 2013). These findings suggested that by middle‐age, the proliferative ability of type A and type C cells is markedly impaired, indicating that the SVZ neurogenic decline is an early event in mice. This impairment of SVZ neurogenic potential was not associated with changes in dopamine transporter (DAT) immunofluorescence in the Str, nor in striatal DA and high affinity synapotosomal DA uptake, or in the number of DAergic cell bodies in the SNpc (L'Episcopo et al., 2013). These findings thus indicated that, besides the nigrostriatal DAergic influence, other factors contributed to SVZ impairment as early as middle‐age.

Aging mice showed an especially‐impaired recovery from MPTP‐induced nigrostriatal histopathological and neurochemical impairment for the entire duration of the study, both at the striatal and SNpc levels (L'Episcopo, Serapide, et al., 2011b; L'Episcopo et al., 2011a, 2011c). In addition, MPTP‐treatment magnified aging‐induced SVZ impairment associated with a failure to recover from SVZ and nigrostrial DAergic injury (Figure S3), in stark contrast with robust SVZ and DAergic post‐injury recovery as observed in younger counterparts (L'Episcopo et al., 2011a, 2012, 2013). Hence, aging and exposure to environmental toxins represent a double hit leading to a marked impairment of NSC survival, proliferation and neuron differentiation capacity, as revealed both in vivo and in vitro*.* However, extending in vitro culturing in the presence of fibroblast growth factor 2 (FGF2) and EGF, can reverse age‐ and MPTP‐induced neurogenic impairment, indicating that the changing SVZ microenvironment with age, coupled to the exposure to harmful factors, may well contribute to the inhibition of SVZ neurogenic potential, and thus we further interrogated whether it is possible to revert such neurogenic impairment.

### 4.2 WβC‐signalling failure mediates aging and MPTP‐induced SVZ impairment in PD

We next addressed whether the age‐dependent dysfunctional status of the SVZ microenvironment might influence the prototypical components of Wnt/β‐catenin pathway in the aging SVZ. Using real‐time PCR we identified that young adult SVZ‐NSCs harbor most Fzds, but *Fzd1* was the most abundant within the transcripts studied. On the other hand, while NSCs from the aged SVZ exhibit small changes in *Fzd* transcript levels, after MPTP challenge a significant decrease in *Fzd1* was observed in aging, but not young, NSCs. In keeping with this result, western blotting showed a sharp Fzd1 down‐regulation only in MPTP‐aged NSCs, indicating that neurotoxin exposure may impair the ability of aged NSCs to respond to Fzd1 ligands. Both aging and neurotoxin exposure exerted a synergic inhibition of canonical WβC activation in SVZ cells, as reflected by decreased *CTNNB1* (β‐catenin) and *AXIN2* transcript levels, associated with a sharp upregulation of *Gsk3b*. Immunohistochemistry showed reduction of β‐catenin*^+^* cells associated with reduced BrdU incorporation in the SVZ of aged compared to younger mice, thereby supporting aging‐induced dysregulation of WβC‐mediated signalling both in the VM (L'Episcopo et al., 2011a) and SVZ (L'Episcopo et al., 2012).

### 4.3 A dysfunctional Wnt/glial inflammatory connection is a key player in SVZ‐NSC disruption in aging and PD

#### 4.3.1 Microglial modulation of NSCs is age‐ and inflammation‐dependent

By the use of a controlled in vitro environment, we next addressed the distinct roles of young and aged microglia. In these ex vivo*/*in vitro cellular models, NSCs derived from young and aged SVZs were cocultured with either young or aged glia, with the aging process ostensibly switching microglia from a neurogenesis‐promoting to a neurogenesis‐inhibitory phenotype. Here, direct coculture of young NSCs with purified microglia significantly influenced neurogenesis as a function of age, with exposure to 2‐day‐old and 2‐month‐old microglia resulting in a sharp increase in NSC proliferation and neuroblast formation. By contrast, 24‐month‐old microglia reduced NSC proliferative potential and the formation of neuroblasts (L'Episcopo et al., 2013). This finding clearly supported the idea that microglial age, and not NSC age, is critical for directing beneficial or harmful effects on NSCs.

This suggestion was corroborated by the use of the nonsteroidal anti‐inflammatory NO‐donating drug, HCT1026, that was previously shown to mitigate microglial activation in aging mice and to protect nigrostriatal DAergic neurons in young, middle aged, and aged PD rodent models in vivo (see L'Episcopo et al., 2011c; L'Episcopo et al., 2013; L'Episcopo, Tirolo, Testa, et al., 2010a; L'Episcopo et al., 2012). Hence, HCT10126 efficiently counteracted both the pro‐inflammatory phenotype and the neurogenesis‐inhibitory effects of old glia, corroborating the harmful neurogenic effects of a microglial proinflammatory environment, as well as the potential to revert and even up‐regulate neurogenesis with NO‐non‐steroidal anti‐inflammatory drug (NSAID) treatment (Figure 4).

#### 4.3.2 Astrocyte‐derived Wnt1 and astrocyte‐microglia interactions shape the response of SVZ‐NSCs to age and MPTP

Astrocyte dysfunctions with age and neurodegeneration are well recognized processes leading to a marked downregulation of astrocyte's supportive, neuroprotective and pro‐neurogenic properties (Barkho et al., 2006; Jiao & Chen, 2008; L'Episcopo, Tirolo, Peruzzotti‐Jametti, et al., 2018a; L'Episcopo, Tirolo, Serapide, et al., 2018b; Marchetti & Abbracchio, 2005; Marchetti et al., 2013; Sousa‐Victor et al., 2018; Yang et al., 2014). Within the SVZ niche, astrocyte dysfunction as a consequence of age and exposure to the PD neurotoxin was shown to inhibit both proliferation and neuroblast formation, as observed both in vivo and in vitro. Studying the age‐dependency of astrocyte's proneurogenic effects, we found that while the direct coculture of young NSCs with 2‐day‐old or 2‐month‐old Str astrocytes sharply increased the percentage of BrdU‐expressing and MAP2a‐expressing NSCs, coculture with aged Str astrocytes decreased both proliferation and neuroblast formation. When young and aged NSCs were treated with CRD‐Fzd1, which inhibits the effects of Wnt ligands that bind to Fzd1, co‐culturing with young Str astrocytes failed to increase NSC neurogenic potential, thereby indicating that, with age, the efficacy of astrocyte‐derived Fzd1 ligands decline (L'Episcopo et al., 2013).

The contribution of Wnt1 was next studied using real‐time PCR analysis. In accordance with our previous studies obtained in mesencephalic astrocytes (L'Episcopo et al., 2011a), we determined *Wnt1* transcripts in Str astrocytes: *Wnt1* expression levels were sharply downregulated in aged Str astrocytes, corroborating previous data uncovering an age‐dependent decline of *Wnt1* in VM astrocytes (L'Episcopo et al., 2011a). Likewise, adult hippocampal neurogenesis that relies on astrocyte‐derived Wnt3a at the SGZ niche (Lie et al., 2005) is significantly inhibited with age, as a consequence of reduced Wnt3a levels and a reduced number of hippocampal astrocytes secreting Wnt3a (Okamoto et al., 2011). Specifically, this reduction in Wnt3a affected the expression of target genes such as *NeuroD1* and retrotransposon L1 (*LINE1*), thereby inhibiting DCX expression and SGZ neurogenic potential (Okamoto et al., 2011). Comparable effects of aging were reported by Miranda et al. in 2012, underscoring that the aged brain microenvironment decreased hippocampal neurogenesis via a disruption of Wnt‐mediated survivin*,* a known mitotic regulator and recognized modulator of WβC‐signalling, which in turn reduced SGZ‐NSC proliferation (Miranda et al., 2012). Accordingly, enhancing Wnt signalling can ameliorate age‐related deficits in cellular and cognitive function (Seib et al., 2013). In the same year, Jang et al. (2013) further reported that a genetic deletion of the endogenous Wnt antagonist sFRP3 stimulated adult hippocampal neurogenesis and promoted antidepressant action (Jang et al., 2013).

Likewise, the studies of Hofmann, McBryan, Adams, and Sedivy (2014) and Orellana et al. (2015) corroborated the decline of Wnt genes with the aging process in mouse tissues and supported the link between age‐related inflammation and WβC‐signalling in the rat hippocampus. As a proof of concept that WβC‐signalling declines with increasing age, Bayod et al. (2015) reported decreased canonical Wnt‐signalling in the hippocampus of a senescence accelerated mouse‐prone 8 (SAMP8) mouse model of accelerated aging. More recently, Cho et al., (2019) documented that inhibition of sFRP3 rescues neural progenitor proliferation in the hippocampal dentate gyrus of BubR1H/H mice (a novel mouse model of accelerated aging), and suggested that the endogenous Wnt inhibitor cooperates with BubR1 function to regulate brain development, myelination, and hippocampal neurogenesis. Finally, Kase et al. (2019) recently reported the involvement of WβC‐signalling in the p38‐induced age‐dependent decline of adult neurogenesis.

Of specific interest, in MPTP‐aged mouse model of PD we found that such an age‐dependent Wnt1 down‐modulation of striatal astrocytes appeared to result from a dysfunctional astrocyte‐microglia crosstalk (see L'Episcopo et al., 2012, 2013, 2014b; L'Episcopo, Tirolo, Peruzzotti‐Jametti, et al., 2018a; L'Episcopo, Tirolo, Serapide, et al., 2018b). Accordingly, exposure of young astrocytes to aged microglia resulted in a sharp decrease of Wnt1 expression. On the other hand, when astrocytes were exposed to young microglia or NO‐NSAID (HCT1026)‐treated aged microglia, a significant increase of *Wnt1* transcript levels was observed, thereby counteracting aging‐induced astrocyte *Wnt1* depletion, which associated to increased SVZ neurogenic potential as revealed both in vivo and in vitro (L'Episcopo et al., 2013).

Together, these informations unveiled that, with the aging process, the exacerbated microglial pro‐inflammatory phenotype can disrupt astrocyte homeostasis at the SVZ niche via a down‐modulation of WβC‐signalling, likely through a dysfunctional astrocyte–microglia interaction (L'Episcopo et al., 2013; L'Episcopo, Tirolo, Peruzzotti‐Jametti, et al., 2018a; L'Episcopo, Tirolo, Serapide, et al., 2018b). Accordingly, pharmacological activation of WβC‐signalling by treatment with the specific GSK‐3β antagonist AR can rescue the impaired NSC potential both in vitro and in vivo (L'Episcopo e al., 2013, 2012; Marchetti & Pluchino, 2013; Figure 4).

#### 4.3.3 Aging and WβC‐signalling at the crossroad of Nrf2‐driven Hmox1‐self‐adaptive response and mitochondrial dynamics

NRF2 is a conserved basic leucine zipper transcription factor which affords cytoprotection against xenobiotics and ROS through induction of antioxidant and electrophile response elements (see Tebay et al., 2015). The beneficial roles of Nrf2‐mediated transcriptional programs in various oxidative stress‐related disease models, such as chronic neurodegeneration, inflammation, carcinogenesis, and pathogenesis associated with environmental toxicant exposure, are well recognized (see Abdalkader, Lampinen, Kanninen, Malm, & Liddell, 2018; Hayes, Chowdhry, Dinkova‐Kostova, & Sutherland, 2015). Indeed, mitochondrial ROS production is regulated by the Nrf2 pathway by controlling mitochondrial bioenergetics. Specifically, Nrf2 has been positively associated with mitochondrial biogenesis through the direct upregulation of mitochondrial transcription factors and is involved in the mitochondrial quality control system through mitophagy activation. Moreover, several mitochondrial proteins participate in regulating Nrf2 to form a reciprocal regulatory loop between mitochondria and Nrf2 (see Ryoo & Kwak, 2018) .

Nrf2 promotes antioxidant and phase II detoxification enzymes, proteasomes, and several transcription factors involved in mitochondrial biogenesis. Hmox1, is a key mediator of cellular adaptive (i.e. antioxidant and anti‐inflammatory) responses (Bitar & Al‐Mulla, 2011; Chen et al., 2009; Song et al., 2006; Surh et al., 2009). This protein is induced by hypoxia, cytokines, and oxidative stress, amongst other factors. Hmox1 is itself an antioxidant protein that protects cells from oxidative damage by downregulating ROS levels. Thus, a deficiency in this Nrf2‐Hmox1 coordinated response may well accompany aging‐associated disorders such as neurodegeneration and cancer. Accordingly, several lines of evidence including in vivo Nrf2‐deficient mouse models, post‐mortem studies of PD brains, and genetic association studies of patients indicate a link between Nrf2 dysregulation and PD pathogenesis (Johnson & Johnson, 2015).

Specifically, astrocytes are central players in Nrf2‐Hmox1 induction following different types of brain insult, including MPTP exposure (Chen et al., 2009). We thus hypothesized that an astrocyte‐driven disbalance of the Nrf2‐Hmox1 axis, resulting from a dysfunctional astrocyte‐microglia crosstalk, might contribute to aging‐induced SVZ impairment (L'Episcopo et al., 2013). Hence, we uncovered a reduced *Nrf2/Hmox1* expression within the aged SVZ niche, compared with younger SVZ counterparts. Of specific interest, while MPTP significantly increased *Hmox1* at a gene expression and protein levels, in both striatal and SVZ‐astrocytes of young mice, aged‐MPTP mice failed to upregulate the Nrf2/Hmox1 axis (L'Episcopo et al., 2013). Significantly, the mRNA transcript of the pivotal mediator of microglial ROS generation, *gp91Phox*, was sharply up‐regulated in the aging SVZ compared with younger counterparts. Moreover, upon MPTP exposure, *gp91Phox*, *Nos2,* and the major proinflammatory (Th1) cytokines, such as *Tnfa*, significantly increased in the SVZ niche, underscoring a microglial switch towards the activated M1 phenotype. These inflammatory mediators, critically involved in inflammation‐dependent DAergic degeneration, associated with the up‐regulation of ameboid‐shaped ionized calcium‐binding adapter molecule 1 (IBA1)^+^ reactive microglia phenotype within the Str bordering the SVZ (L'Episcopo et al., 2013). The critical importance of *Nrf2* on rat hippocampal NSC function was recently underscored by Corenblum et al. (2016) using RNA interference and overexpression assays, as well as in *Nrf2* knockout mice, as shown by Ray et al., (2018). This emphasizes the importance of *Nrf2* during the middle‐age time period also for hippocampal SGZ neurogenic modulation, corroborating the reduced expression of *Nrf2* as an important mechanism mediating the age‐related neurogenic decline.

Conjunctly, recent data also identified the activity of the mitochondrial electron transport chain and oxidative phosphorylation machinery as a critical determinant of adult hippocampal neurogenesis (Beckervordersandforth et al., 2017). Here, perturbation of mitochondrial complex function by ablation of Tfam reproduces multiple hallmarks of aging in hippocampal neurogenesis, whereas pharmacological enhancement of mitochondrial function ameliorates age‐associated neurogenesis defects, thereby “identifying mitochondrial function as a potential target to ameliorate neurogenic dysfunction in the aging hippocampus” (Beckervordersandforth et al., 2017). Interestingly, Tfam down‐modulation as promoted by MPTP/MPP^+^ DAergic neurotoxins (Wang et al., 2014), or genetic knockdown (Wen et al., 2019), also associates a with down‐regulation of downstream WβC‐signalling genes (L'Episcopo et al., 2011c, 2012, 2013; Wang et al., 2014; Wen et al., 2019), corroborating Wnt signalling crosstalk with major NSC/neuronal self‐protective pathways (Arrázola, Silva‐Alvarez, & Inestrosa, 2015).

Together, these results may suggest that the aging‐dependent mitochondrial dysfunction in synergy with neurotoxin exposure may negatively impact on the astrocytic *Nrf2*‐driven *Hmox1* response within the SVZ niche in vivo. Remarkably, this process, resulting from an age‐dependent dysregulation of astrocyte‐microglia interactions, can contribute to the exacerbated oxidative and inflammatory SVZ status and the decline of astrocyte Wnt‐dependent regulation, ultimately leading to NSC neurogenic impairment and loss of SVZ plasticity.

From the bulk of the summarized results, it is tempting to speculate that in addition to governing the redox balance with in the SVZ niche, the *Nrf2*‐induced *Hmox1* target gene may simultaneously protect astrocytes, there by upregulating the expression of vital Wnt signalling elements that switch‐on key components required for maintaining SVZ cells in a proliferative state, promoting differentiation, and/or exerting neuroprotective effects (Figure 4).

## 5 ADDRESSING WNT/β‐CATENIN SIGNALLING IN PD NEUROGENESIS

### 5.1 Harnessing Wnt signalling targeting the inflammatory *Nrf2/Hmox1* axis restores SVZ neurogenesis and promotes DAergic neurorestoration in PD

The development of anti‐inflammatory drugs targeting inflammatory molecules to preserve adult neurogenesis during PD neurodegeneration‐induced inflammation has been addressed by an increasing number of studies (see L'Episcopo, Tirolo, Serapide et al., 2018b; Peruzzotti‐Jametti, et al., 2018a). The studies of Worlitzer et al. (2012) showed that pharmacological inhibition of neuroinflammation in the 6‐OHDA mouse model by the semi‐synthetic tetracycline derivative minocycline led to increased NSC proliferation in the SVZ (Worlitzer et al., 2012). Also, in the 6‐OHDA minocycline‐treated group the proportion of neuroblasts that had migrated deeply (50 μm and more) into the lesioned Str was increased 2–4‐fold compared to all other groups, however no newly generated striatal neurons could be detected (Worlitzer et al., 2012). Four months after surgery, in the presence of minocycline therapy, the authors observed increased oligodendrogenesis in the lesioned Str which was associated with a significant behavioral improvement in PD symptoms, thus indicating oligodendrocytes and oligodendrogenesis as having an important role to play in the pathology of PD (Worlitzer et al., 2012). In our own studies (L'Episcopo et al., 2012) we addressed the potential of anti‐inflammatory drug treatment in the modulation of SVZ neurogenesis in MPTP mice, and its ability to reverse WβC down‐modulation both in young and aging mice, using the mixed cyclooxygenase inhibitor HCT1026. HCT1026 is endowed with a favourable safety profile and shown to downregulate microglial activation via the inhibition of PHOX and iNOS‐derived RNS, resulting in a significant protection of nigrostriatal DAergic neurons in PD rodent models, in both young and aging mice (L'Episcopo, Tirolo, Caniglia, et al., 2010b; L'Episcopo et al., 2011c; L'Episcopo, Tirolo, Testa, et al., 2010a). HCT1026 efficiently increased β‐catenin protein and β‐catenin^+^ staining in SVZ of HCT1026‐MPTP mice of both ages back to normal untreated control levels in the face of a sharp downregulation of active GSK‐3β, as compared to MPTP mice fed with a control diet. These effects of HCT1026 were associated with a significant increase in the number of BrdU^+^ and DCX^+^ expressing cells in the striatal SVZ, correlating with a strong inhibition of reactive microglial cell density in the Str and SVZ (L'Episcopo et al., 2013). Treatment of aging mice with HCT1026 resulted in a severe down‐regulation of microglial pro‐oxidant and pro‐inflammatory mediators in the SVZ, including *Nos2* (iNOS) and *TNFα,* and a reversal of MPTP‐induced up‐regulation of inflammatory mRNA species, as opposed to aged mice fed with a control diet. This effect was accompanied by the normalization of redox/inflammatory balance in aged‐HCT1026 mice, as revealed by a robust up‐regulation of *Nrf2* and *Hmox1* transcripts associated with microglial inhibition, thus confirming HCT1026‐induced up‐regulation of an SVZ anti‐inflammatory response. Furthermore, a significant degree of DAergic re‐innervation followed HCT1026 treatment of MPTP mice, in line with a significant protection of midbrain cell bodies, confirming our previous report (L'Episcopo, Tirolo, Caniglia, et al., 2010b; L'Episcopo et al., 2011c).

Finally, we uncovered a crosstalk between Wnt/β‐catenin pathway and the pivotal phosphoinositide 3‐kinase (PI3K)/protein kinase B (Akt)/GSK‐3β signalling cascades, that finely control the transcriptional activator β‐catenin, which in turn represents a point of convergence to direct proliferation/differentiation/survival in the SVZ stem niche. The “rejuvenation” of the SVZ may have beneficial consequences for DAergic neuroprotection, and vice versa (L'Episcopo et al., 2013).

Targeting Nrf2 as a promising therapeutic avenue in neurodegeneration has been recently reviewed in the work of Abdalkader et al. (2018), underscoring its important role in modulating neurodegenerative processes, including its ability to suppress mitochondrial dysfunction, likely underlying its crucial role in efficiently protecting and driving an anti‐stress response within the major neurogenic niches.

Coupled with the previous sections, evidence may suggest exogenous activation of β‐catenin signalling, or pharmacological mitigation of a microglial proinflammatory phenotype up‐regulating β‐catenin in the SVZ, successfully rescues NSC proliferation and neuroblast formation. These findings implicated modulation of the Wnt/β‐catenin/Nrf2/Hmox1 axis, either directly or indirectly via inflammation‐dependent SVZ modulation, as a potential strategy for DAergic neuroprotection/self‐repair (Marchetti & Pluchino, 2013) (Figure 4).

### 5.2 The midbrain tegmental aqueduct as a novel Wnt‐dependent NSC‐DAergic niche

Studies of the last few years have identified the Aq‐PVR as a novel Wnt/β‐catenin responsive brain region and addressed the properties of Aq‐PVR‐NSCs in MPTP‐induced PD (L'Episcopo et al., 2014a). Looking at the factors/mechanisms regulating the behaviour of these Aq‐PVR‐NSCs in vitro, we addressed the neurogenesis‐promoting and inhibitory conditions during the process of aging and MPTP‐induced nigrostriatal injury, investigating the potential to activate these progenitors to rescue DAergic plasticity in aged‐MPTP mice. To this end, we also took advantage of transgenic BATGAL mice expressing nuclear beta‐galactosidase under the control of the β‐catenin‐activated transgene (BAT) promoter (Maretto et al., 2003), together with both in vivo experimental PD young and older mice models, coupled to ex vivo*/*in vitro cell cultures of midbrain‐Aq‐PVR‐NSCs (mNSCs).

### 5.3 Proliferation and neuron differentiation of mNSCs in vitro depends upon age and MPTP

We first characterized mNSC properties during in vitro clonal expansion supporting their neurogenic potential as indicated by the expression of proliferation, precursor, pro‐neural, and astrocyte cell markers, and very low forebrain (distal‐less homeobox 2, *Dlx2*) and hindbrain (homeobox D3, *Hoxd3*) markers, as opposed to the high expression of the midbrain marker engrailed 2 (*En2*) (L'Episcopo et al., 2014a). We next addressed the changes of mNSC response as a function of age and PD, by assessing the proliferative, differentiation and survival properties of mNSCs from 2–5 (young, Y) and 9–12 (aged, A) month‐old mice, in basal conditions and during MPTP‐induced injury and recovery. As observed in SVZ‐NSCs, we then showed that with the aging process, the percentages of both BrdU^+^ incorporation and Tuj1^+^ (β‐III‐tubulin) cell formation significantly decreased (L'Episcopo et al., 2014a). While Y‐mNSCs isolated 7 days post‐MPTP treatment responded with only a transient decrease of their proliferation and neuronal differentiation potential that returned back to pre‐MPTP levels by 45 days post‐treatment, in A‐mNSCs MPTP did not change the low levels of A‐mNSC proliferation and neuron formation after in vitro expansion.

These findings, coupled to the increased Caspase‐3‐like activity of A‐ as compared to Y‐counterparts, suggested reduced mNSC survival capacity as a result of aging and MPTP, underscoring reduced mNSC plasticity, a phenomenon correlating with the failure of TH^+^ neurons to recover from MPTP insult.

### 5.4 Loss of astrocyte‐dependent WβC‐signalling with age dictates loss of mNSC neurogenic potential in PD

#### 5.4.1 In vitro studies

**Figure 5:**
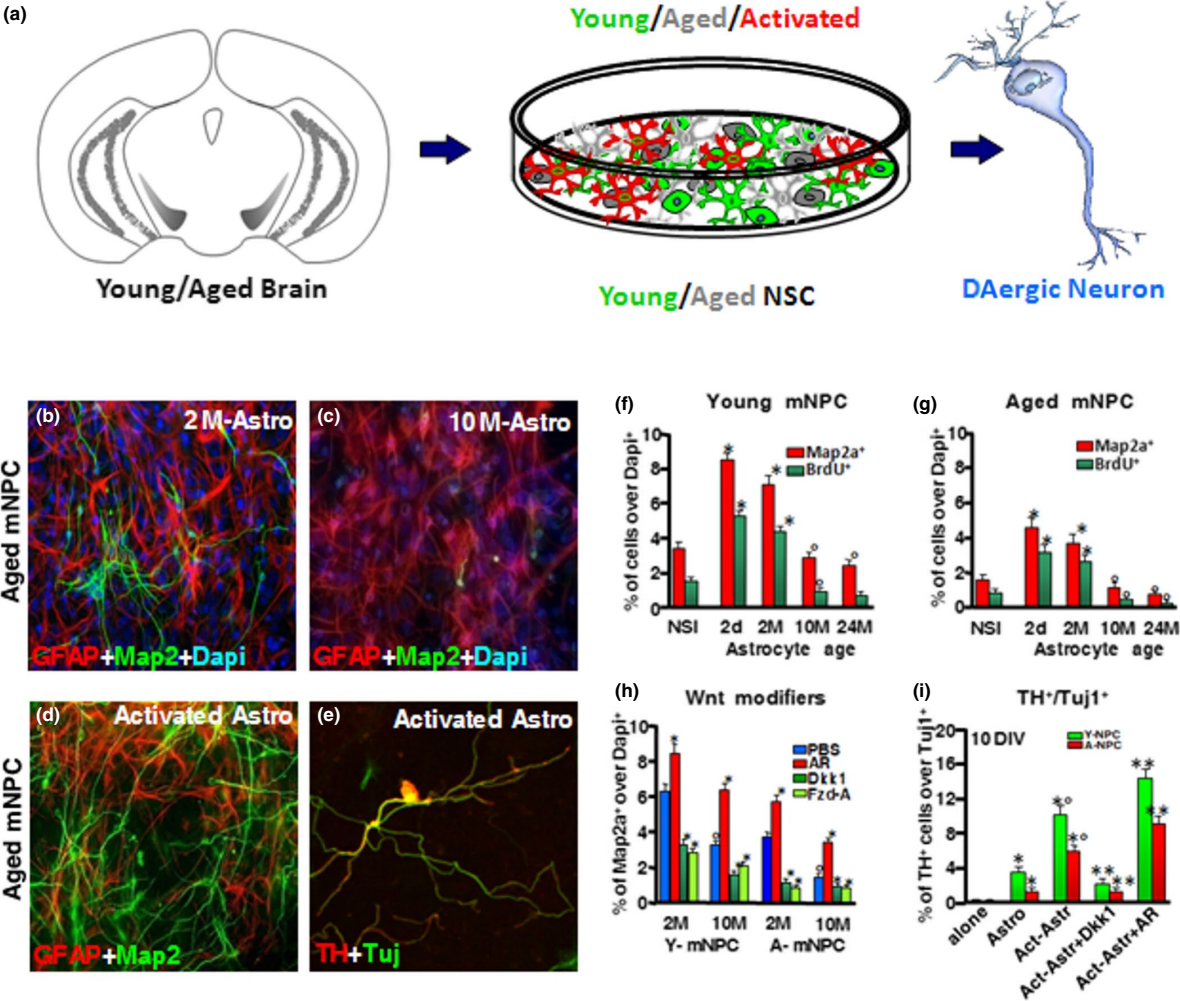
Astroglial age inhibits canonical WβC*‐*signalling: rescue effect of astrocyte Wnt1. (a) scheme of co‐culture between young (Y)/aged (A)/activated astrocyte (Astro) and Y/A midbrain‐Aq‐PVR‐NSCs (mNSCs). (b–e) representative confocal images of dual staining with GFAP (red) and Map2a (green) with Dapi (blue) counterstain comparing A‐mNSCs co‐cultured with 2‐ (B) and 10‐ (C) month‐old astro, or activated astro (C), respectively. Dual staining with TH (red) and Tuj1 (green) is illustrated in a representative confocal image in A‐ mNPC cocultured with activated astro (D). (f–i) percent of BrdU and Map2a expressing cells in cocultures of Y‐ (F) or A‐ (G) mNPC cocultured with 2d, 2M, 10M or 24 M astro. The GSK‐3β antagonist, AR efficiently counteracted the inhibitory effect of aged astro in both Y/A‐NPCs (H). Conversely, Wnt/β‐catenin antagonistm with Dikkopf‐1 (Dkk1), or Frizzled‐1‐cysteine rich domain (Fzd‐A), reversed young astro effects (H). Activated astro promote TH^+^ neuron formation in both Y‐and A‐mNPCs after 10 DIV in differentiating medium (i). Exposure to AR further increases while Dkk1 inhibits TH^+^ formation (I). *p < .05 versus. NSI; °*p* < .05 versus. 2d and 2M astro in Y‐(E) and A‐mNPC (F), respectively. *p < .05 versus. PBS and °*p* < .05 versus. 2 M and 10 M astro (H); *p < .05 versus Y‐A‐NPC cultured alone, °*p* < .05 versus. Y‐/A‐NPC with untreated astro; *p* < .01 versus. Y‐/A‐NPCs without Dkk1 or AR (I)

By analogy with our studies carried out at the SVZ level, we observed reduced canonical WβC‐ signalling in Aq‐PVR‐NSCs as a function of age and MPTP injury (L'Episcopo et al., 2014a). Given our previous studies documenting a robust pro‐neurogenic effect of midbrain astrocytes (L'Episcopo et al., 2011a), coupled to the effect of glial age in SVZ‐NSCs (L'Episcopo et al., 2013), we then used different co‐culture paradigms between Y/A Aq‐PVR astrocytes and Y/A‐ mNSCs, with or without WβC agonists or antagonists to verify the effect of glial age and glial‐derived Wnts. We found that: (a) Wnt/β‐catenin signalling is dysfunctional in aged mNSCs; (b) astrocytes of the tegmental midbrain lose their pro‐neurogenic and DAergic differentiation abilities via a decline of astrocyte‐derived factors including Wnts; and (c) astrocyte‐coculture paradigms coupled to Wnt‐activation regimens can rescue A‐NSCs and promote TH^+^ neuron formation (Figure 5).

#### 5.4.2 In vivo studies

The tegmental Aq which is closed to DAergic cell bodies was shown to respond to MPTP‐induced DAergic neuron death with an extraordinary astrocyte‐dependent remodeling and activation of radial glia. A remarkable increase of polysialylated neural cell adhesion molecule‐positive (PSA‐Ncam^+^) cells was observed, suggesting NSC activation early upon DA neuron injury, but this phenomenon was absent in aging mice denoting the failure to recover from MPTP injury (L'Episcopo et al., 2014a). Hence, while MPTP‐induced DA neuron death promoted an early and remarkable astrocyte‐dependent remodeling associated to WβC‐signalling activation in Nurr1^+^ post‐mitotic DA precursors, in surviving and repairing SNpc‐DA neurons of young mice, this phenomenon was absent upon aging. Radial glia‐like progenitors express *Wnt1* (Bonilla et al., 2008), while deletion of *Wnt1* induces a severe loss of radial glia‐like cells and DA neurons (Arenas, 2014; Bonilla et al., 2008; Prakash & Wurst, 2014; Tang et al., 2009, 2010). Especially, DA neuron precursors within the developing human mesencephalon show radial glial characteristics (Hebsgaard et al., 2009). The key role of WβC‐signalling was demonstrated by Briona, Poulain, Mosimann, and Dorsky (2015), showing its requirement for radial glial neurogenesis following spinal cord injury.

Wnt signalling via β‐catenin promotes the differentiation of Nurr1^+^/TH^‐^ DAergic precursors. By contrast, removal of β‐catenin in DAergic progenitors reduces the progression from committed DAergic progenitors to DAergic neurons (Joksimovic & Awatramani, 2014; Prakash and Wurst, 2014; Tang et al., 2009, 2010). Conversely, inhibition of GSK‐3β blocks the degradation of β‐catenin, thus the abundance of DAergic neurons increases through conversion of precursors expressing Nurr1 into TH^+^ neurons (see Arenas, 2014; L'Episcopo et al., 2014a; Toledo et al., 2017). We then hypothesized that the changing properties of the aging midbrain‐Aq microenvironment negatively impacted the neurogenic potential of Aq‐mNSCs on WβC‐signalling. Using β‐catenin reporter mice, expressing nuclear β‐galactosidase as a readout of β‐catenin activation, we next uncovered a dramatic loss of active Wnt signalling in the Aq‐PVRs of aged‐MPTP mice (L'Episcopo et al., 2014a).

## 6 THERAPEUTIC MODULATION OF WNT/β‐CATENIN SIGNALLING

### 6.1 Harnessing astrocyte‐dependent WβC‐signalling by pharmacological inhibition of GSK‐3β to restore mNSC DAergic potential in PD

**Figure 6:**
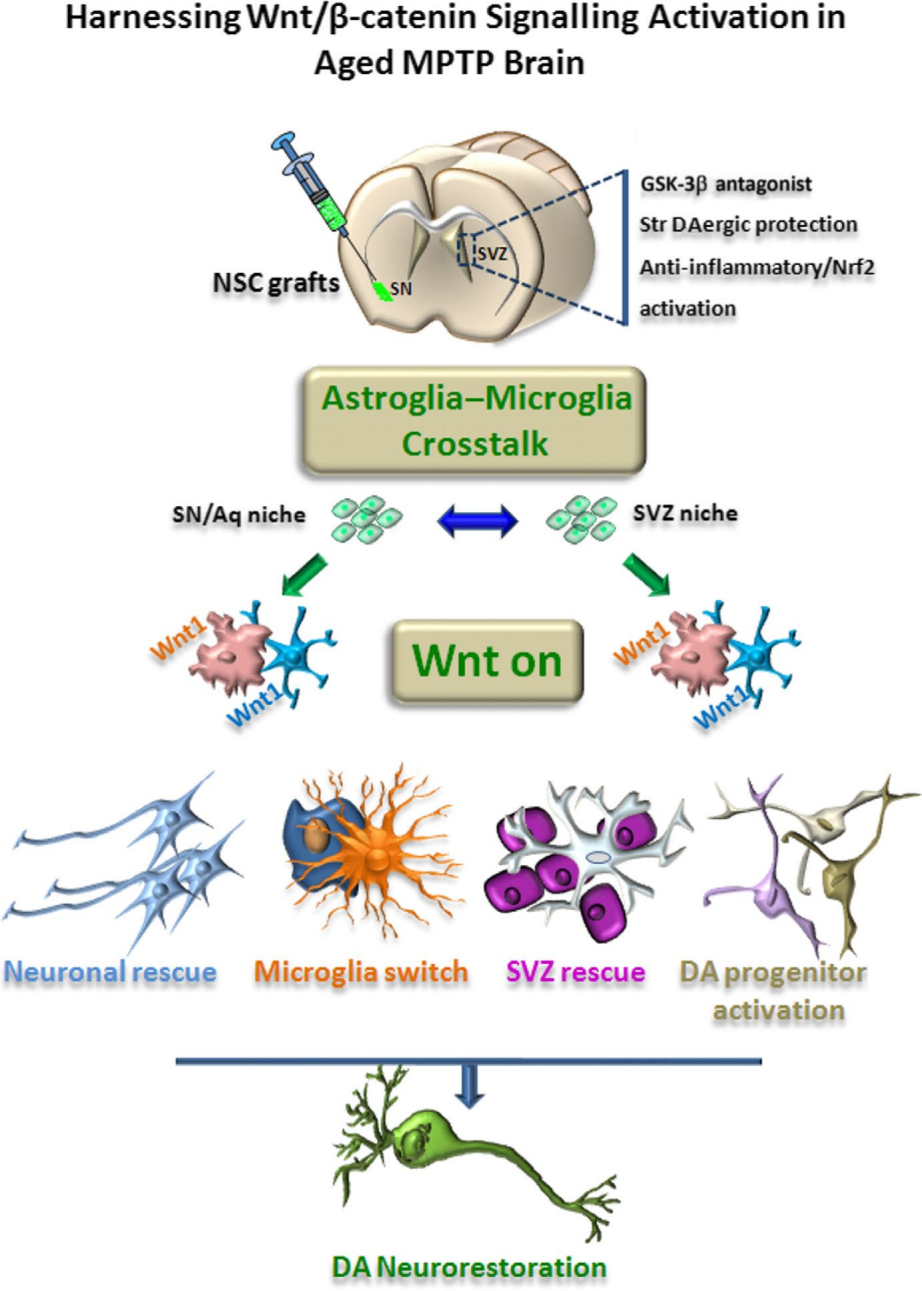
Harnessing WβC‐signalling activation in the aged, inflammed PD brain. Schematic representation of the ‘*Wnt on*' neurorestoration instructed by grafted NSCs in the aged PD brain. With age, the inflammed midbrain microenvironment coupled to dysfunctional astrocyte‐microglia interactions and environmental toxin exposure (MPTP) inhibit active Wnt‐signalling in astrocytes (“Wnt‐off” condition), resulting in exacerbation of inflammation and inhibition of Wnt‐dependent neuroprotective and pro‐regenerative capacities of astrocytes with harmful consequences for mDA neuron survival and repair from MPTP injury. NSCs, NSC‐derived astrocytes and endogenous astrocytes switch the inflammatory/Wnt‐genetic cascade via astrocyte‐neuron and astrocyte‐microglia crosstalk both at the SNpc and Aq‐PVR DA niche levels. Reciprocally, astrocyte derived Wnt1 further influence both exogenous and endogenous NSCs, reduce microglia pro‐inflammatory status, thus favouring beneficial effects for an overall TH neurorescue (“Wnt on”) program. Exogenous pharmacological treatments rescuing the impaired SVZ neurogenis (including GSK‐3β antagonists, anti‐oxidant and anti‐inflammatory drug treatment and DAergic activation promoting SVZ neurogenic rescue) are also illustrated

We thus addressed the potential to overcome aging‐induced loss of Wnt1‐mediated activation of mNSCs in the Aq‐PVR, by pharmacologically targeting this niche in situ via intracerebral injection of the GSK‐3β‐antagonist AR: direct WβC‐signalling activation in aged‐MPTP mice resulted in a marked stimulation of proliferation and differentiation of Aq‐PVR‐Nurr1^+^/TH^‐^ precursors into fully differentiated TH^+^/Nurr1^+^ DAergic neurons, thus contributing to both the histopathological and functional restoration of nigrostratal DAergic neurons (L'Episcopo et al., 2014a) (Figure 6). Recently, using Nestin‐CreER^TM^::ROSA26‐LacZ mice and a CD133‐Promoter2‐Cre plasmid construct in 6‐OHDA intranigral injected mice, Xie et al. (2019) traced the origin of SN newborn DAergic neurons. The authors showed a significant increase in the SVZ‐derived NSCs of the third ventricle (3V) and Aq‐surrounding regions. Hence, the SN newborn DAergic neurons were mainly derived from the migration and differentiation of the NSCs in the SVZ of 3V‐ and Aq‐adjacent regions (Xie et al., 2017), thus supporting our indication that quiescent neuroprogenitors of mNSCs can be activated not only in vitro (L'Episcopo et al., 2011c), but more importantly, in vivo*,* upon SNpc‐DAergic lesions with PD neurotoxins (L'Episcopo et al., 2014b).

After our first studies demonstrating dysregulated WβC‐signalling in the MPTP mouse model of PD, both in vivo and in vitro (L'Episcopo et al., 2011a;b), more recent reports corroborated the key role of WβC in the physiopathology of PD (see Marchetti, 2018 for an up‐to‐date review). Among others, Singh, Mishra, Bharti, et al. (2018b) used short hairpin (sh)RNA‐lentiviruses to genetically knockdown *AXIN2* to up‐regulate WβC‐signalling in the SNpc, in 6‐OHDA‐induced parkinsonism rats. Not surprisingly, genetic knockdown of *AXIN2* (a WβC‐pathway suppressor, see Section 2), up‐regulated WβC‐signalling with beneficial consequences for DAergic neurogenesis (Singh, Mishra, Bharti, et al., 2018b).

Coupled to the bulk of results obtained in vivo in the two β‐catenin reporter lines (L'Episcopo et al., 2014b; L'Episcopo, Tirolo, Peruzzotti‐Jametti, et al., 2018a), and in conjunction with literature findings, these studies documented and corroborated that in young mice the MPTP‐induced DA neuron death (a) activates endogenous Wnt/β‐catenin signalling in the Aq‐PVR niche; (b) β‐catenin is highly expressed in postmitotic Nurr1^+^/TH^‐^ neurons with an ongoing β‐catenin signalling activation both in Nurr1^+^/DAT^‐^ pre‐DAergic and recovering DAT^+^ neurons during the neurorescue process of young MPTP mice; and (iii) it is possible to reactivate the aged Aq‐PVR niche via both in situ infusion of Wnt/β‐catenin activators or nigral NSC transplantation (L’Episcopo et al., 2011a).

### 6.2 Harnessing astrocyte‐dependent WβC‐signalling by NSC transplantation within the aged MPTP‐lesioned SNpc

Earlier studies on PD mouse models first established that NSCs “possess an intrinsic capacity to rescue dysfunctional neural pathways” via multiple homeostatic effects (see Madhavan, Daley, Paumier, & Collier, 2009; Madhavan, Daley, Sortwell, & Collier, 2012; Madhavan, Ourednik, & Ourednik, 2008; Ourednik et al., 2009; Redmond et al., 2007). Several studies carried out in rodents and non‐human primate models of PD have shown the ability of transplanted NSCs to survive, proliferate, migrate and produce functional effects via the production of neuroprotective, neurogenic, angiogenic, and anti‐inflammatory factors in the PD brain. Notably, NSCs grafts can also establish neurogenic niches in nonneurogenic regions, and are capable to generate neurons and glia, thus improving the microenvironment, the plasticity and function of the aged hippocampus (Shetty & Hattiangady, 2016).

However, very little is known on NSC ability to promote neuroprotection in the aged PD brain. Recently, using SVZ‐derived adult NSCs transplanted into the aged MPTP‐injured SN, we showed a remarkable time‐dependent endogenous nigrostriatal DAergic neurorestoration program orchestrated by astrocyte‐derived Wnt1 (L'Episcopo, Tirolo, Serapide, et al., 2018b). Here, within the aged microenvironment, NSC grafts survived and proliferated within the SN of both sides in situ. A significant fraction of transplanted cells acquired an astrocytic phenotype both at the SN level and at the midbrain Aq‐PVRs, with a robust migration of NSCs to the Wnt‐sensitive midbrain DAergic niche. NSC grafts in the aged SNpc promoted the expression of *Wnt1* in both NSC‐derived and reactive endogenous astrocytes (RAS), leading to Fzd1/β‐catenin signalling in SNpc‐TH^+^ neurons. NSC grafts did not change the mRNA levels for a number of growth and neurotrophic factors, including glial cell‐derived growth factor (*Gdnf*)*,* insulin‐like growth factor 1 (*Igf1*)*,* nerve growth factor (*Ngf*)*,* transforming growth factor α (*Tgfa*)*, Tgfb* and *Fgf8*. By contrast, several DAergic‐specific developmental factors including *En1/En2*, and the direct WβC‐signalling targets, *Lef1*, LIM Hhomeobox transcription factor 1 α (*Lmx1a)* and *Fgf20,* as well as the indirect Wnt1/β‐catenin targets pituitary homeobox 3 (*Pitx3*) and brain‐derived neurotrophic factor (*Bdnf*) (see Zhang et al., 2015, and references therein), were up‐regulated of about 2.5–3.5‐fold in MPTP + NSC grafts versus MPTP + PBS sham controls, with *Wnt1* being the most up‐regulated DAergic‐specific neurodevelopmental and pro‐survival factor amongst those studied (L'Episcopo, Tirolo, Serapide, et al., 2018b). Likewise, looking at 19 WβC‐signalling members, the canonical Wnts components were sharply down‐regulated in the aged MPTP brain, whereas major Wnt‐antagonists, including the *Dkk* and *sFrp* families of Wnt inhibitors, and *Gsk3b* showed marked up‐regulation of 6‐to‐14‐fold in aged‐MPTP tissues. In stark contrast, NSC grafts, again, reversed the MPTP‐induced up regulation of *Dkks*, *sFrps* and *Gsk3b* (L'Episcopo, Tirolo, Serapide, et al., 2018b).

At the Aq‐PVRs, such an up‐regulation of WβC‐signalling promoted the accumulation of β‐catenin‐immunofluorescence and β‐catenin^+^/DAPI^+^ cells, as unveiled in BATGAL/NSC‐grafted MPTP mice (L'Episcopo, Tirolo, Serapide, et al., 2018b). Here, astrocyte‐derived *Wnt1* expression recapitulated a genetic *Wnt1*‐dependent DAergic developmental programme inciting the acquisition of the mature Nurr1^+^/TH^+^ neuronal phenotype in post‐mitotic DAergic precursors, thus increasing the endogenous pool of midbrain DAergic neurons contributing to the reversal of MPTP‐induced nigrostriatal toxicity (L'Episcopo, Tirolo, Serapide, et al., 2018b). As a functional outcome of this Wnt‐mediated Aq‐PVR neuroprogenitor activation, TH^+^ cell bodies with different morphologies and size were seen coursing from the Aq‐PVR and along the midline down to the ventral tegmental area (VTA) of NSC grafted mice, mimicking TH^+^ neurons trafficking from the Aq to the SNpc observed in younger MPTP mice during SNpc neurorepair (L'Episcopo et al., 2014a; L'Episcopo, Tirolo, Serapide, et al., 2018b).

Corroborating the role of WβC‐signalling in Dkk1‐treated NSC‐grafted MPTP mice, we observed no TH^+^ neurons in Aq‐PVRs. These data, thus, demonstrated that NSC grafts activate β‐catenin transcription, inducing a marked increase of TH^+^ cells within the Aq‐PVR and VTA, whereas this process is blocked by WβC‐antagonism, finally resulting in the inhibition nigrostriatal histopathological and functional repair (L'Episcopo, Tirolo, Serapide, et al., 2018b).

Together, these novel findings supported the potential of nigrostriatal restoration by activating WβC‐signalling in Wnt‐responsive niches, through either pharmacological and cellular approaches aimed at activating/recruiting endogenous progenitors and rescuing the imperiled/diseased DAergic neurons. Moreover, these data suggested that WβC‐signalling activation by NSC grafts at the SNpc and Aq‐PVR is required for NSC‐promoted DAergic functional restoration of aged PD mice. Thus, harnessing WβC‐signalling represents a potential means to boost the endogenous self‐repair/regenerative capacity of the aged PD brain (Figure 6).

### 6.3 Up‐regulating adult neurogenenesis as a disease modifying strategy for PD: is WβC‐ signalling the common denominator?

**Figure 7:**
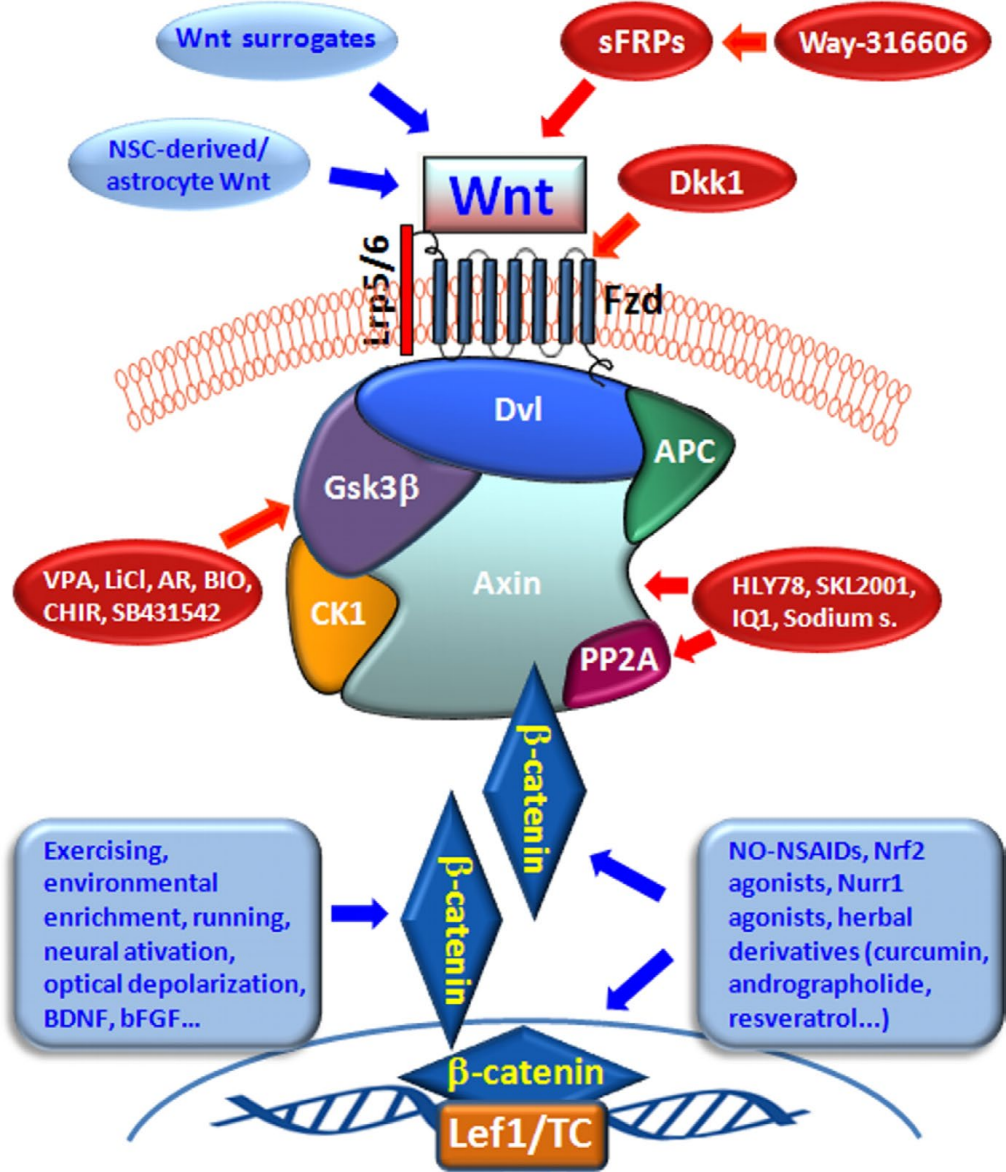
WβC‐signalling activation as a disease modifying strategy for PD. Schematic illustration of potential interventions targeting either indirectly or directly WβC‐signalling activation (WβC‐AC) in the central nervous system. As shown in the diagram WβC‐AC can be targeted at different steps (for details see the text and Tables 1 and 2). Indirect WβC‐AC, in blue, include several physical conditions/manipulations/pharmacological treatments, such as environmental enrichment, physical exercise, optogenetic and neural stimulation, or stem cell therapies, the administration of various classes of molecules, including anti‐oxidant and anti‐infammatory molecules (NO‐NSAIDs), Nrf2‐ and Nurr1‐agonists or herbal derivatives (curcumin, andrographolide, resveratrol). Direct activation of canonical Wnt/Fzd/β‐catenin cascade with: *Fzd‐LRP5/6 Wnts surrogates*; SVZ‐NSC‐derived astrocyte's *Wnt1*, in blue; small Wnt activators targeting *Axin‐LRP6* (HLY78, SKL2001), *PP2A* agonists, in red (IQ1, Sodium selenite), directly interacting with multiple components of β‐catenin destruction complex; GSK‐3β antagonists, in red (VPA, LiCl, AR, BIO, CHIR, SB431542); or endogenous antagonists such as secreted FZD‐related proteins, sFRPs (WAY310666), in red. bFGF, basic fibroblast growth factor; casein kinase 1α (CK1α); Dishevelled, Dvl; Fzd, frizzled; GSK‐3β, glycogen synthase kinase 3β;NO‐NSAID, nitric oxide‐releasing non‐steroidal anti‐inflammatory drugs; Nrf2, nuclear factor erythroid 2‐related factor 2; NSC, neural stem/progenitor cell; Nurr1, nuclear receptor related 1 protein; sFRP, secreted Frizzled‐related proteins; SVZ, sub‐ventricular zone; tumor suppressor adenomatous polyposis coli, APC; WβC‐AC, Wnt/β‐catenin signalling activation

Different conditions, cell therapies and/or pharmacological treatments are being studied for their potential to modulate endogenous neurogenesis to favour neuroprotection, neurorepair and immunomodulation (Wenker & Pitossi, 2019), with an increasing number of manipulations being associated to either a direct or indirect ability to up‐regulate the WβC‐signalling cascade (Tables 1 and 2, Figure 7). Owing to its crucial function in stem cell maintenance and tissue homeostasis, there are potential risks and concerns for modulation of WβC‐signalling, regarding both the safety and selectivity, underscoring “Dr. Jekyll'‐Mr Hyde's” dual facet of WβC‐signalling activation/inhibition (Banerjee et al., 2019; Chen et al., 2019; Herrera‐Arozamena, Martí‐Marí, Estrada, Fuente Revenga, & Rodríguez‐Franco, 2016; Huang, Tang, et al., 2019a; Huang, Yan, et al., 2019b; Janda et al., 2017; Kahn, 2014; Mahmood, Bhatti, Syed, & John, 2016; Maiese, 2015; Narcisi et al., 2016; Nusse & Clevers, 2017). Therefore, indirect WβC modulation appears as an attractive strategy to up‐regulate endogenous neurogenesis. Environmental enrichment, physical exercise, the administration of hormones, anti‐oxidant and anti‐inflammatory molecules, pharmacological, pharmacogenetic and/or epigenetic strategies, optogenetic and neural stimulation, or deep brain stimulation, and stem cell therapies, are all being explored for their potential to reverse neurogenic impairment, incite neurorepair and/or reverse cognitive impairment, apparently through activation of WβC signalling, in experimental models of NDs. Also, a certain number of herbal derivates, primarily from the Traditional Chinese Medicine, endowed with pharmacological properties (including anti‐cancer, anti‐bacterial, and anti‐oxidant activities) are increasingly being studied for their ability to modulate WβC‐signalling (recently reviewed by Liu D et al., 2019), with interesting therapeutic potentials for NDs including PD (Table 1 and 2 and Figure 7).

**Table 1:** Interventions indirectly targeting WβC‐signalling activation (WβC‐AC)‐targeted in the central nervous system

Intervention	Outcome	References
Modulation of inflammation and oxidative stress		
NO‐NSAID (in vitro/in vivo)	NO‐NSAID counteracts MPTP‐induced decrease in proliferation and neuronal differentiation potential, and age/MPTP‐induced downregulation of the Nr2‐Hmox1 axis in aged SVZ NSCs via increased **WβC‐AC**	L'Episcopo et al. (2013), L'Episcopo et al. (2012), L'Episcopo et al. (2011a), L'Episcopo et al. (2011c), L'Episcopo et al. (2010b)
L‐NAME (in vitro)	Counteraction of decrease in aged SNpc‐DAergic neuroprotection; striatal DAergic re‐innervation; amelioration of motor deficit via **WβC‐AC**	
Apocynin (in vitro)	L‐NIL and apocynin counteract NO and oxidative stress, promote SVZ‐NSC proliferation and neuronal differentiation	
Modulation of Nrf2‐Hmox1 axis		
KMS99220	Novel morpholine derivative activates Nrf2; orally active in MPTP models, ameliorating degeneration and motor deficit; *WβC‐AC involvement to be elucidated*	Lee et al. (2018)
Proinflammatory cytokines/chemokines		
Chemokines (CCL3, CXCL10, CXCL11), in vitro	Chemokine pretreatment of VM astrocytes and aged astrocytes upregulate Wnt1 expression, promoting neurogenesis and DAergic neurogenesis from adult NSCs and inducing neuroprotection against MPTP/MPP^+^‐induced injury via **WβC‐AC**	L'Episcopo et al. (2011a), L'Episcopo et al. (2014a), L'Episcopo et al. (2018a)
Tetracyclines		
Minocycline, in vivo	Counteracts TNF‐α‐induced decrease in neurogenesis in 6‐OHDA model of PD; *WβC‐AC involvement to be elucidated*	Worlitzer et al. (2012)
Herbal derivatives		
Curcumin (from the rhizome of turmeric), in vivo	Counteracts bisphenol‐induced inhibition of hippocampal neurogenesis via **WβC‐AC**	Tiwari et al. (2019)
	Protects against oxidative stress‐induced injury in a rat model of PD via **WβC‐AC**	Wang, et al. (2017)
	Ameliorates cognitive function, enhances neurogenesis, mitigates inflammation and mitochondrial dysfunction in hippocampus in a rodent model of gulf war illeness; *WβC‐AC involvement to be elucidated*	Kodali et al. (2018)
Exercise, environmental enrichment	Physical activity and environmental enrichment regulate the generation of neural precursors in the adult mouse substantia nigra; *WβC‐AC involvement to be elucidated*	Klaissle et al. (2012)
	Endurance exercise promotes neuroprotection against MPTP injury via enhanced neurogenesis, antioxidant capacity and autophagy; *WβC‐AC involvement to be elucidated*	Jang, kwon, Song, Cosio‐Lima and Lee (2018a), Jang et al. (2018b)
Neural activation	Neural activity‐induced **WβC‐AC** up‐regulates expression of BDNF.	Zhang, Zhang, Deng, et al., 2018a
Neurotrophic factors	BDNF promotes growth of neurons and NSCs, possibly through activation of the PI3K/**GSK‐3β/β‐catenin** pathway	Li et al. (2017)
	BDNF promotes human neural stem cell growth via **GSK‐3β‐mediated crosstalk with the WβC pathway**	Yang et al. (2016)
Optical depolarization	Optogenetic activation of VM astrocytes enhances DAergic differentiation of NSCs and promotes brain repair in PD rodent models; *WβC‐AC involvement to be elucidated*	Yang et al. (2014)
	Optical depolarization of DCX‐expressing neuroblasts promotes cognitive recovery and maturation of newborn neurons after traumatic brain injury via **WβC‐AC**	Zhang, Huang, et al. (2016c)
	Coupling of optogenetics and light‐sheet microscopy reveals **WβC‐AC** during embryogenesis and post‐natal development	Kaur et al. (2017)
	**WβC‐AC** can be controlled in vivo via light responsive capsules.	Ambrosone et al. (2016)
	Optical depolarization promoted the maturation of neural stem cells via **WβC‐AC**	Xia et al. (2014)
Electromagnetic fields	Enhanced olfactory memory in mice exposed to extremely low frequency electromagnetic fields via **WβC‐AC**‐induced modulation of SVZ‐neurogenesis	Mastrodonato et al. (2018)
Transcription factors		
Nurr1 agonists; amodiaquine (AQ), pharmacological stimulation	AQ stimulates Nurr1's transcriptional function, enhancing adult hippocampal neurogenesis; *WβC‐AC involvement to be elucidated*	Kim et al. (2016)
	Pharmacological stimulation of Nurr1 induces neuroprotection and anti‐inflammatory effects in the 6‐OHDA PD‐model; *WβC‐AC involvement to be elucidated*	Smith et al. (2015)
Nanoparticles (Liposomes)		
Curcumin liposomes	Curcumin‐loaded nanoparticles promote adult neurogenesis and reverse cognitive deficits in an Alzheimer's Disease model via **WβC‐AC**	Tiwari et al. (2017)
Paclitaxel liposomes	Collagen microchannel scaffolds carrying paclitaxel‐liposomes induce neuronal differentiation of NSCs through **WβC‐AC** in spinal cord injury repair	Li et al. (2018)

Note

6‐OHDA, 6‐hydroxydopamine; BDNF, brain‐derived neurotrophic factor; DA, dopamine; DCX, doublecortin; GSK‐3β, glycogen synthase kinase 3β; Hmox1, heme oxygenase 1; L‐NAME, N_ω_‐Nitro‐L‐arginine methyl ester hydrochloride; MPP^+^, 1‐methyl‐4‐phenylpyridinium; MPTP, 1‐methyl‐4‐phenyl‐1,2,3,6‐tetrahydropyridine; NO‐NSAID, nitric oxide‐releasing non‐steroidal anti‐inflammatory drugs; Nrf2, nuclear factor erythroid 2‐related factor 2; NSC, neural stem/progenitor cell; Nurr1, nuclear receptor related 1 protein; PI3K, phosphoinositide 3‐kinase; SNpc, substantia nigra pars compacta; SVZ, sub‐ventricular zone; TNF‐α, tumor necrosis factor alpha; VM, ventral midbrain; WβC‐AC, Wnt/β‐catenin signalling activation.

Bold is used to highlight WβC connection

**Table 2:** Interventions directly targeting WβC‐signalling activation (WβC‐AC)‐targeted in the central nervous system

Intervention	Outcome	References
Modulation of Fzd/β‐catenin/Nurr1		
Wnt1, Wnt3a, Wnt5a	**Wnt1**, **‐3a**, and **‐5a** expression is differentially regulated during development. **Wnt3a** promoted the proliferation of precursor Nurr1^+^ cells. **Wnt‐1 and ‐5a** increased the number of rat midbrain DAergic neurons in E14.5 precursor cultures. **Wnt‐1** increased the proliferation of Nurr1^+^ precursors, up‐regulated cyclins D1 and D3, and down‐regulated p27 and p57 mRNAs	Castelo‐Branco et al. (2004)
	**Wnt5a** increases differentiation of midbrain DAergic cells and phosphorylation of dishevelled	Schulte et al. (2005)
	VM glia express region‐specific transcription factors and regulate DAergic neurogenesis through **Wnt5a** secretion	Castelo‐Branco et al. (2006)
	**Wnt5a**‐treated midbrain NSCs improve DA cell replacement therapy in parkinsonian mice	Parish et al. (2017)
	**Wnt1** and astrocyte‐derived **Wnt1** promote proliferation and neuron differentiation of adult SVZ‐NSCs	L'Episcopo et al. (2011a)
	Astrocyte‐derived **Wnt1** and chemokine‐primed aged astrocytes promote proliferation and DAergic differentiation of Aq‐PVR‐NSCs via **WβC‐AC**	L'Episcopo et al. (2014a)
Modulation of Axin‐LRP5/6		
Phenanthridine derivatives (HLY78 and novel molecules)	HLY78 targets the DIX domain of Axin and potentiates the Axin‐LRP6 association, thus promoting LRP6 phosphorylation and transduction of **WβC**‐signalling	Wang et al. (2012)
	Identification of structure‐activity relationship‐optimized phenanthridine derivatives as new WβC‐AC pathway agonists	Chen et al., 2019
N‐(3‐(1H‐imidazol‐1‐yl)propyl)‐5‐(furan‐2‐yl)isoxazole‐3‐carboxamide (SKL 2001)	Protective role in in vivo cytotoxicity models	Huang, Tang, et al., 2019a
Wnt surrogates	Water‐soluble Fzd‐Lrp5/6 heterodimerizers consisting of Fzd5/8‐specific and Fzd‐reactive binding domains, endowed with a **WβC‐AC** potential through ligand‐induced receptor heterodimerization. Promote growth in a broad range of primary human organoid cultures in a fashion comparable to Wnt3a. Exhibit Wnt activity in vivo	Janda et al. (2017)
Modulation of protein phosphatase 2A (PP2a)		
Sodium selenite IQ1	Interacts with Axin, APC, and β‐catenin; identified as target of Wnt agonist/IQ and sodium selenite; activates **WβC‐AC** via increased activation of β‐catenin and decreased GSK‐3β levels in a triple transgenic mouse model of Alzheimer's disease	Jin et al. (2017)
Inhibition of sFRPs		
N‐substituted piperidinyl diphenylsulfonyl sulphonamides (e.g. WAY‐316606)	Modulators of Wnt signalling through inhibition of secreted frizzled‐related protein I (sFRP‐1)	Moore et al. (2009), Bodine et al. (2009), Warrier et al. (2007)
Gene KO	sFRP‐mediated Wnt sequestration represents a potential therapeutic target for Alzheimer's disease. sFRP3 inhibition improves age‐related cellular changes in BubR1 progeroid mice	Cho et al. (2019)
Inhibition of ADP ribosylation factor GTPase activating protein 1 (ARFGAP1)		
QS11	QS11 analogues act as small Wnt molecule synergist; direct inhibition of enzymatic activity of purified ARFAP1 protein and cellular activation of the **WβC**‐pathway confirm the direct inhibition of ARFGAP1 by QS11 and also suggest the presence of other potential cellular targets of QS11	Jin et at al., 2017
GSK‐3 antagonists		
Indirubin‐3‐monoxime, kenpaullone	GSK−3β inhibition/β‐catenin stabilization in ventral midbrain precursors increases differentiation into DAergic neurons via **WβC‐AC**	Castelo‐Branco et al. (2006), Reviewed by Arenas (2014), Toledo et al. (2019)
GSK‐3β siRNA	GSK−3β siRNA treatment of NSCs from MPTP‐injured mice resulted in a significant increase in the percentage of cells expressing BrdU associated with an increased percentage of MAP2a^+^ cells via **WβC‐AC**	L'Episcopo et al. (2011b), L'Episcopo et al. (2012)
CHIR99201, SB‐216763, SB‐415286	CHIR99201 is a substituted aminopyrimidine derivative that potently and selectively inhibits GSK−3 in vitro and *in viv*o	Ring et al. (2003)
	SB−216763 and SB−415286 are cell‐permeable, structurally distinct maleimides that potently and selectively inhibit GSK−3	Coghlan et al. (2000)
	Midbrain floor plate precursors are derived from hPSCs in 11 days following exposure to small molecule activators of sonic hedgehog and **WβC‐AC**. Enrichment for canonical Wnt signalling upon CHIR99021 treatment. Induction and neurogenic conversion of hESC‐derived midbrain floor plate precursors is dependent on CHIR99021 addition	Kriks et al. (2011)
	Establishes a means of obtaining a scalable source of FOXA2^+^/TH^+^ neurons for neural transplantation, a major step on the road towards considering a cell‐based therapy for PD	
	CHIR99201 counteracts the altered differentiation potential of Gaucher's disease iPSC neuronal progenitors due to Wnt/β‐catenin downregulation by **WβC‐AC**	Awad et al. (2017)
	GSK−3 inhibition via CHIR99021 known to promote proliferation of neuroprogenitors by activating **β‐catenin** and Notch‐related cell cycle genes in the presence of bFGF and EGF, increased neural differentiation.	Esfandiari et al. (2012)
	CHIR99201‐mediated **WβC‐AC** exploited in a step‐by‐step protocol for generation of regionally specified neural progenitors and functional neurons from human embryonic stem cells under defined conditions.	Kirkeby et al. (2012)
	Generation of VM DA progenitors and mature VM DA neurons from hPSCs under defined, xeno‐free conditions onvolving the use of CHIR99201. DA cells are primed for clinical translation, capable of correcting motor asymmetry in 6‐OHDA lesioned rats: significant increases in the yield of appropriately specified OTX2/FOXA2‐expressing progenitors and FOXA2/TH DA neurons; elevated DA metabolism and functional electrophysiological properties reflective of mature VM DA neurons	Niclis et al. (2016)
	Combined treatment of *trans‐*retinoic acid with CHIR 99,201 significantly enhanced neurogenesis via **WβC‐AC**	Nierode et al. (2012)
Ro3303544	Selective and potent maleimide inhibitor of GSK−3β that increases the number of newborn neurons in the olfactory bulb via **WβC‐AC** when administered systemically or via stereotaxic icv	Adachi et al. (2007)
N‐(4‐Methoxybenzyl)‐N′‐(5‐nitro‐1,3‐thiazol−2‐yl)urea (AR‐A014418)	Specific and potent inhibitor of GSK−3β with neuroprotective effects	Bhat et al. (2003)
	Counteracts SNpc cell death in murine PD models after systemic treatment via **WβC‐AC**	Wang et al. (2013)
	Counteracts MPTP‐induced neurotoxicity upon systemic or icv administration via **WβC‐AC**	L'Episcopo et al. (2011a;b)
	Counteracts MPTP‐induced inhibition of SVZ‐NSC proliferation and neuron differentiation in young and aged mice via **WβC‐AC**	L'Episcopo et al. (2012)
	Counteracts aging and MPTP‐induced nigrostriatal degeneration of aged mice via **WβC‐AC**	L'Episcopo et al. (2013)
	Counteracts age and MPTP‐induced decrease Aq‐PVR‐NSC proliferation and activates pre‐DAergic Aq‐PVR Nurr1^+^/TH^‐^ precursors after intracerebral injection around the Aq‐PVRs and increased Nurr1^+^/TH^+^ neurons in the SNpc, via **WβC‐AC**	L'Episcopo et al. (2014a)
	Results in decreased tau phosphorylation and hippocampal neuron death and decreased proinflammatory cytokines in transgenic mouse models of Huntington disease	L'Episcopo et al. (2016)
6‐bromoindirubin‐3′‐oxime (BIO)	Selective inhibitor of Tyr216/276 phosphorylation of GSK−3; maintains pluripotency in human and mouse embryonic stem cells via **WβC‐AC**	Sato et al. (2016)
	Stimulates post‐stroke neurogenesis, neuroblast migration to the ischemic cortex, neuronal differentiation and functional recovery after ischemic stroke via **WβC‐AC**	Wang et al. (2014)
Valproic acid	Has been recently shown to increase β‐catenin levels and to induce the expression of NeuroD1, a Wnt target gene involved in neurogenesis in the hippocampus of 3xTg‐Alzeimer's disease model mice via **WβC‐AC**	Zeng et al. (2019)
Lithium chloride (LiCl)	Non‐specific antagonism of GSK−3 modulates a panel of signalling pathways	Clément‐Lacroix et al. (2005)
	Synergy between NSC transplantation and systemic LiCl‐mediated GSK−3β antagonism promoted recovery upon spinal cord injury via **WβC‐AC**	Zhang, Zhang, Deng, et al. (2018a)
L807mts	Peptide GSK−3β inhibitor reduces inflammation and promotes neuroprotection and behavioural recovery in Alzheimer's disease model mice; *WβC‐AC involvement to be elucidated*.	Licht‐Murava et al. (2016)
TWS119	A 4,6‐disubstituted pyrrolopyrimidine GSK−3β inhibitor that induces neurogenesis, based on counting of TuJ1 positive cells with correct neuronal morphology	Ding et al. (2003) Huang, Tang, et al. (2019a)
Herbal derivatives		
Andrographolide	As a competitor of GSK−3β, stimulates neurogenesis in the adult hippocampus via increased **WβC‐AC**	Varela‐Nallar et al. (2015)
Ilexonin A	Chinese medicine component; neuroprotective during ischemic injury via **WβC‐AC**	Zhang, Zheng, Yang et al. (2016a)
Resveratrol	Protects SAMP8 brain under metabolic stress, ameliorating mitochondrial function and activating **WβC‐signalling** Counteracts neurodegeneration and abnormal neurogeneis in a rodent model of status epilepticus via suppression of inflammation; *WβC‐AC involvement to be elucidated*	Palomera‐Avalos et al. (2017), Mishra et al. (2015)
Tricin	Modulates **WβC‐AC** by upregulating Wnt3a expression and downregulating GSK−3β expression	Zhang and Li (2018)
Gedunin (from seeds of Azadirachta indica)	Inhibits neuroinflammation arising from Aβ−_1−42_ oligomer exposure in a microglial cell line via the activation of the Nrf2 anti‐inflamatory and anti‐oxidant axis, *WβC‐AC involvement to be elucidated*	Tom et al. (2019)
Flavonoids (e.g. quercetin, taxifolin), curcumin	Modulate proliferation, differentiation, growth and apoptosis via direct/indirect **WβC‐AC**. *See also *Table 1	Sivrastava and Sivrastava (2019), Mohana et al. (2018), Razak et al. (2018)
Ginsenoside Rb1	A derivative of traditional Chinese medicine ginseng; metabolite shown to promote **WβC‐AC**	Zhou et al. (2018), Reviewed by Liu et al. (2019)

Note

3xTg, APP/PS1/Nestin‐GFP triple transgenic mice; 6‐OHDA, 6‐hydroxydopamine; APC, adenomatous polyposis coli; Aq‐PVR, mesencephalic aqueduct‐periventricular region; bFGF, basic fibroblast growth factor; DA, dopamine; EGF, epidermal growth factor; FOXA2, forkhead box A2; Fzd, frizzled; GSK‐3β, glycogen synthase kinase 3β; hESC, human embryonic stem cell; hPSC, human pluripotent stem cell; icv, intracerebroventricularly; iPSC, induced pluripotent stem cell; KO, knockout; Lrp, low‐density lipoprotein receptor‐related protein; MAP2A, microtubule‐associated protein 2a; MPTP, 1‐methyl‐4‐phenyl‐1,2,3,6‐tetrahydropyridine; NSC, neural stem/progenitor cell; Nurr1, nuclear receptor related 1 protein; OTX2, orthodenticle homeobox 2; PD, Parkinson's disease; SAMP8, senescence accelerated mouse‐prone 8; sFRP, secreted Frizzled‐related proteins; SNpc, substantia nigra pars compacta; SVZ, sub‐ventricular zone; TH, thyrosine‐ hydroxylase; VM, ventral midbrain; WβC‐AC, Wnt/β‐catenin signalling activation.

#### 6.3.1 Micro‐ and nanocarriers

Micro/nanoparticulate delivery vehicles may be engineered to release multiple biomolecules with spatio‐temporal control are being developed, aimed at mobilizing NSCs efficiently from their niches to promote their engraftment at lesioned areas, or creating a long term anti‐inflammatory microenvironment (Li et al., 2018; Riabov et al., 2017; Tiwari et al., 2014; Zhang, Zhang, Deng, et al., 2018a; Zhang, Shi, et al., 2018b; Zhang, Zhang, Yang, et al., 2018c; Zhang, Huang, et al., 2016c). Curcumin‐loaded nanoparticles powerfully induce adult neurogenesis and reverse cognitive deficits in an Alzheimer's disease model via the activation of WβC‐signalling pathway (Tiwari et al., 2015, 2019). The ability of curcumin treatment to ameliorate cognitive and mood function was previously associated with increased neurogenesis as well as mitigation of inflammation and mitochondrial dysfunction in the rodent hippocampus (Kodali et al., 2018).

Additionally, loading paclitaxel (PTX)‐encapsulated liposomes into a collagen microchannel scaffold, leading to a prolonged sustained release of PTX, was recently shown to provide an instructive microenvironment for neuronal differentiation of NSCs, motor and sensory neuron regeneration, axon extension and beneficial functional outcomes via activation of the WβC‐signalling pathway (Li et al., 2018). Conversely, inorganic nanocarriers such as silver nanoparticles have been attributed with negative effects, reportedly disrupting β‐catenin signalling and resulting in reduced neurite lengths in differentiating NSCs (Cooper et al., 2019).

#### 6.3.2 Optogenetics

Optogenetics is a novel approach allowing specific cell stimulation by external illumination which may remotely manipulate intracellular pathways in single cells (Zhang et al., 2016a,b,c), using channelrhodopsin‐2 (ChR2) activation to allow cationic currents to depolarize genetically targeted cells. Yang et al. (2014), found that optogenetic activation of VM astrocytes can enhance the DAergic differentiation of stem cells and promote brain repair in PD models in vivo and in vitro, very likely via the mediation of WβC activation. Kaur et al. (2017) addressed the potential of coupling optogenetics and light‐sheet microscopy to study Wnt signalling during embryogenesis, showing that WβC‐signalling is required not only for *Drosophila* pattern formation, but also for maintenance later in development. Additionally, Zhang, Huang, et al. (2016c) reported that optical depolarization of DCX‐expressing cells induced cognitive recovery and maturation of newborn neurons after traumatic brain injury via WβC‐signalling pathway activation. WβC‐signalling also plays a key role in controlling neuron activity‐regulated neurotrophic factor (i.e. *Bdnf*) expression (Zhang, Zhang, Deng, et al., 2018a; Zhang, Shi, et al., 2018b; Zhang, Zhang, Yang, et al., 2018c). Also, in human NSCs, Yang et al. (2016) showed the ability of BDNF to promote their growth via GSK‐3β‐mediated crosstalk with the WβC‐ signalling pathway, and Li et al. (2017) involved the contribution of the PI3K/Akt/GSK‐3β/β‐catenin pathway in BDNF‐induced neuron and NSC growth. Ambrosone et al. (2016) documented the ability of optogenetic stimulation to promote cell proliferation and the migration of SVZ neuroblasts into the peri‐infarct cortex, asssociated with increased neuronal differentiation and improvement of long‐term functional recovery after stroke. Finally, Mastrodonato et al. (2018) reported enhanced olfactory memory in mice exposed to extremely low frequency electromagnetic fields via WβC‐dependent modulation of SVZ neurogenesis.

### 6.4 Small molecules modulating WβC proteins

In recent years, druggable molecular targets and signalling pathways involved in neurogenic processes have been identified, and as a consequence, different drug types have been developed and tested in neuronal plasticity. The field of small molecules as potential tools to selectively activate or inhibit WβC is increasingly recognized with a number of both established and novel modulators, including Wnt3a‐like agonists, siRNAs and inhibitors targeting GSK‐3β, Axin‐LRP5/6 or transcription factor complexes (Table 2, Figure 6). The manipulation of WβC‐signalling has become an attractive strategy to ameliorate in vitro differentiation protocols for increasing the fraction of midbrain DAergic neurons (see Kirkeby et al., 2017; Toledo et al., 2017; Brodski et al., 2019).

#### 6.4.1 Nurr1 activation

An important potential mediator of WβC‐signalling activation is Nurr1, a nuclear receptor acting as an intracellular transcription factor recognized to contribute to the proliferation and differentiation of NSCs, both during development and in the adult brain. A number of studies documented the importance of Nurr1 and WβC/Nurr1 signalling pathways in promoting neurogenesis from DAergic precursors (reviewed in Arenas, 2014; Toledo et al., 2017; Section 2 and section, sectionprevious sections). Several studies have reported the contribution of Nurr1 in the modulation of cognitive functions (see Kim et al., 2015; Kim et al., 2016, and refs therein). Specifically, the pharmacological stimulation of Nurr1 was shown to improve cognitive functions via the enhancement of hippocampal neurogenesis (Kim et al., 2015, 2016), very likely via up‐regulation of WβC‐signalling. Hence, Kim et al. (2015) identified two antimalarial drugs, amodiaquine (AQ) and chloroquine that stimulated the transcriptional function of Nurr1. Remarkably, these compounds were able to enhance the Nurr1‐dependent transcriptional activation of DAergic‐specific genes. Moreover, they further enhanced transrepression of neurotoxic proinflammatory gene expression in microglia (Kim et al., 2015). Of specific interest, pharmacological stimulation of Nurr1 causes both neuroprotection and anti‐inflammatory effects in the 6‐OHDA lesion model of PD (Kim et al., 2015; Smith et al., 2015). Additionally, these compounds significantly improved behavioral deficits in 6‐OHDA lesioned rat model of PD without any detectable signs of dyskinesia‐like behavior (Kim et al., 2015), underscoring the potential of small molecules targeting Nurr1 as neuroprotective strategy for PD (Kim et al., 2015). In another study, the anti‐malarial AQ powerfully enhanced adult hippocampal neurogenesis, increasing learning and memory processing via a direct neurogenic action of Nurr1 (Kim et al., 2016), as supported by immunocytochemical and immunohistochemical analyses, both in vivo and in vitro (Kim et al., 2016). In addition to its effects on proliferation and differentiation of NSCs, AQ‐treated mice showed a significant enhancement of both short‐ and long‐term memory in the Y‐maze and the novel object recognition test. Together these data suggest that activation of Nurr1 may enhance cognitive functions by increasing adult hippocampal neurogenesis and also indicate that Nurr1 may be used as a therapeutic target for the treatment of memory disorders and cognitive impairment observed in NDs.

#### 6.4.2 Axin‐LRP6

Modulation of the Axin‐LRP6 axis has been the focus of several studies. In 2013, by screening a synthetic chemical library of lycorine derivatives, Wang et al. identified 4‐ethyl‐5‐methyl‐5,6‐dihydro‐[1,3]dioxolo[4,5‐j]phenanthridine (HLY78) as an activator of the WβC‐signalling pathway, via modulation of the Axin‐LRP5/6 interaction. HLY78 targets the DIX domain of Axin and potentiates the Axin‐LRP6 association, thus promoting LRP6 phosphorylation and WβC‐signalling transduction. The authors also identified the critical residues on Axin for HLY78 binding and showed that HLY78 may weaken the autoinhibition of Axin (Wang et al., 2013). Further results have been recently gathered by Chen et al. (2019) on the design, synthesis and structure‐activity relationship for optimization of phenanthridine derivatives as new WβC‐signalling pathway agonists, including evidence for a protective role of the Wnt‐agonists in in vivo cytotoxicity models (Huang, Tang, et al., 2019a; Huang, Yan, et al., 2019b). Recently, protein phosphatase‐2A (PP2A), a multi‐subunit serine/threonine phosphatase that positively regulates the Wnt pathway, has been shown to directly interact with Axin, APC, and β‐catenin, and thus identified as a target of the Wnt agonist/IQ and sodium selenate (Jin et al., 2017). Another possible avenue by which to up‐regulate WβC is to target sFRP‐mediating Wnt sequestration. Unlike the effect of the Dkk family of Wnt‐antagonists on AD, the sFRP molecules have a more pleiotropic impact on the Wnt signalling cascade and may have an important involvement in neurodegeneration. Recently, the potential role of sFRPs on neurodegeneration, their likely involvement, and potential implications in treatment modalities of AD has been reviewed, and future studies will further define sFRPs as potential therapeutic modulators of WβC activation in a number of NDs including PD (Cho et al., 2019; L'Episcopo, Tirolo, Serapide, et al., 2018b; Warrier et al., 2016).

#### 6.4.3 Fzd‐LRP5/6 heterodimerizers

A novel class of WβC‐agonists are the Fzd‐LRP5/6 heterodimerizers (Janda et al., 2017), called “surrogate Wnt‐agonists”. The water‐soluble Fzd‐LRP5/6 heterodimerizers, consist of “Fzd5/8‐specific and Fzd‐reactive binding domains”, endowed with a WβC‐signalling activating potential through ligand‐induced receptor heterodimerization, showed to promote a characteristic β‐catenin signalling response in a Fzd‐selective manner, including the growth of a broad range of primary human organoid cultures, in a fashion comparable to Wnt3a (Janda et al., 2017). The ability of these compounds to be systemically expressed and exhibit Wnt activity in vivo was demonstrated, suggesting a potential “new avenue to facilitate functional studies of WβC‐signalling” (Janda et al., 2017).

#### 6.4.4 GSK‐3 β‐antagonism

A great number of studies indicates that GSK‐3β acts as a key regulator in neural development, including neuroblast generation/migration, neuroprogenitor homeostasis, neural induction, neuronal polarization, axon growth/guidance, and synaptic plasticity (Jope et al., 2017). The activation of GSK‐3β has a role in the phosphorylation of microtubule‐associated protein tau (MAPT), triggering cytoskeleton destabilization, Tau aggregation and neuronal dysfunction or death (Beurel, Grieco, & Joper, 2015; Jope et al., 2017; L'Episcopo et al., 2016). As recalled in previous sections, both earlier and more recent reports indicate that GSK‐3β inhibitors can promote adult neurogenesis both under normal and injury conditions, either in vivo or in vitro. In vitro protocols that modulate Wnt signalling to improve cell therapies for PD are increasingly being developed (Arenas, 2014; Broski et al., 2019; Joksimovic & Awatrami, 2014; Kirkeby et al., 2012, 2017; Kriks et al., 2011; Parish & Thompson, 2014; Prakash & Wurst, 2014; Toledo et al., 2019; Zhang et al., 2015). To date, a number of GSK‐3β‐antagonists have been described, some of which have been tested for their potential to promote adult neurogenesis (Table 2). Amongs others, CHIR is a small molecule used to promote neuroprogenitor homeostasis and neural induction. CHIR was shown to restore WβC‐signalling and to rescue DAergic differentiation in iPSC‐derived NSCs from Gaucher's disease patients, exhibiting developmental defects due to downregulation of canonical WβC‐signalling (Awad et al., 2017). Recently, Nierode et al. (2019) reported high‐throughput identification of factors promoting neuronal differentiation of human neural progenitor cells in microscale 3D cell cultures. In this study, the authors looked at a microarray chip‐based platform to screen the individual and combined effects of 12 soluble factors on the neuronal differentiation of a human neural progenitor cell line (ReNcell VM) encapsulated in microscale 3D Matrigel cultures, revealing that the combined treatment of *trans*‐retinoic acid with CHIR significantly enhanced neurogenesis, while simultaneously decreasing astrocyte differentiation (Nierode et al., 2019).

The cell permeable selective inhibitor of GSK‐3β inhibitor, BIO, is derived from *Tyrian purple indirubins*, that selectively inhibits the phosphorylation of GSK‐3β at Tyr216/276 (Sato et al., 2004) and is widely used to activate Wnt signalling. Recent studies addressing the effect BIO during the sub‐acute and chronic phases after ischemic stroke showed its ability to stimulate post‐stroke neurogenesis, neuroblast migration to the ischemic cortex, neuronal differentiation and functional recovery after ischemic stroke (Wang et al., 2017). Valproic acid has been recently shown to increase β‐catenin levels and to induce the expression of NeuroD1, a Wnt target gene involved in neurogenesis in the hippocampus of 3xTgAD mice (Zeng et al., 2019).

### 6.5 Concluding remarks and future perspectives

Harnessing endogenous pro‐neurogenic mechanisms to counteract the decline of adult neurogenesis and promote DAergic plasticity in the aged brain represents a major goal in the PD physiopathological field. We herein discussed the critical role of WβC‐signalling in rebuilding a regenerative microenvironment and in promoting neuronal differentiation of endogenous or exogenous NSCs, which is pivotal for the recovery of neurologic functions in the aged PD brain.

There is compelling evidence that WβC‐signalling plays a vital role in adult neurogenesis in PD, with the important collaboration of glial pathways. Hence, astrocyte‐derived Wnt1 and WβC activation can engage DAergic neurogenesis/neurorepair in the affected PD brain, as uncovered in transgenic β‐catenin reporter PD‐injured mice. An early downregulation of Wnt signalling in endogenous niches starts by middle age, which is associated to down‐regulation of NSC proliferation and differentiation. Moreover, an up‐regulation of the endogenous sFRP and Dkk families of Wnt‐antagonists, together with the overexpression of the pivotal β‐catenin destruction complex protein, GSK‐3β, in synergy with crucial risk factors, particularly inflammation and MPTP exposure, act in concert to impair Wnt signalling both inside and outside the neurogenic niches, with harmful effects for adult neurogenesis and nigrostriatal neurorestoration. Hence, aged NSCs face a harmful niche microenvironment, and its exacerbation upon MPTP‐induced PD, as a result of a failure of the “*WβC‐inflammatory connection”* which provides a robust homeostatic regulatory mechanism for NSC survival, proliferation, differentiation and integration.

Herein we provided an overview of the molecular mechanism(s) underlying the crosstalk between Wnt‐signalling and NSCs involving astrocyte and microglial mediators, with a crucial role of astrocytic‐microglia crosstalk, including modulation of the Nrf2/Hmox1/WβC‐axis, critical for protection against exacerbated inflammation, oxidative stress and mitochondrial dysfunction. As WβC‐signalling also plays a prominent role in hippocampal SGZ neurogenesis, and in light of the marked decline of WβC‐signalling components in the aged hippocampus, a comparable “*WβC‐inflammatory (dis)connection”* might well be at play in PD‐injured SGZ‐niche.

Remarkable potential exists to revert some of these age‐dependent changes, targeting WβC‐signalling components either directly or indirectly. Pharmacological and cellular therapies, in particular NSC‐grafts, and immunomodulation were documented to ameliorate the aged microenvironment, promoting endogenous neurogenesis, and ultimately boosting a neurorestoration program in the aged PD brain via the up‐regulation of WβC‐signalling.

A number of novel molecules and conditions were reviewed for their potential to activate WβC for translational applications in regenerative medicine. Hence, targeting Axin‐LRP6 with phenanthridine derivatives, PP2A modulators, sFRP inhibitors, and surrogate Wnt‐agonists, showed their ability to promote cell survival/protection and/or immunomodulation. Also, targeting GSK‐3β with small inhibitors, such as CHIR or BIO, can promote neuroprogenitor homeostasis and neural induction, and restore WβC‐signalling in iPSC‐derived neuronal progenitor cells from PD patients. Emerging studies at the interface between NSC biology and tissue engineering are being exploited for innovative therapeutic applications in brain repair/regeneration therapies, including optogenetics, neural stimulation, and micro‐and nanocarriers releasing multiple biomolecules involved in WβC activation. These interventions are aimed at mobilizing NSCs efficiently from their niches, and, in combination with sustained release of therapeutic agents, can be envisaged as a promising approach to induce neuronal differentiation of NSCs, down‐regulating the exacerbated pro‐inflammatory microenvironment and promoting neurorepair in the injured aged PD brain.

In conclusion, the continuous investigations and further deepening of our knowledge on WβC‐signalling and its role on endogenous adult stem cell biology, NSC crosstalk within the PD injured microenvironment, the response of NSCs to different pharmacological/cellular strategies, as well as its implication will translate into therapeutic breakthroughs and novel applications. Specifically, harnessing their synergistic interactions may lead to the optimization of cell‐based therapies for PD.

## CONFLICT OF INTEREST

No conflict of interest to declare.

## AUTHOR CONTRIBUTIONS

Authors contributing to the presented experimental findings and Ms editing: B.M.: conception and design, data analysis and interpretation, manuscript writing; MFS, CT, FL, SC, NT: performed experiments, data analyses and interpretation, final approval of manuscript; JAS, SP: experimental design, data analyses and interpretation, provision of study material, Ms editing, final approval of manuscript.
