# Supplementary figures and images for: Parkinson's disease, aging and adult neurogenesis: Wnt/β‐catenin signalling as the key to unlock the mystery of endogenous brain repair
Source: Aging Cell. 2020 Feb 12;19(3):e13101. doi: 10.1111/acel.13101 (PMC7059166; doi:10.1111/acel.13101)

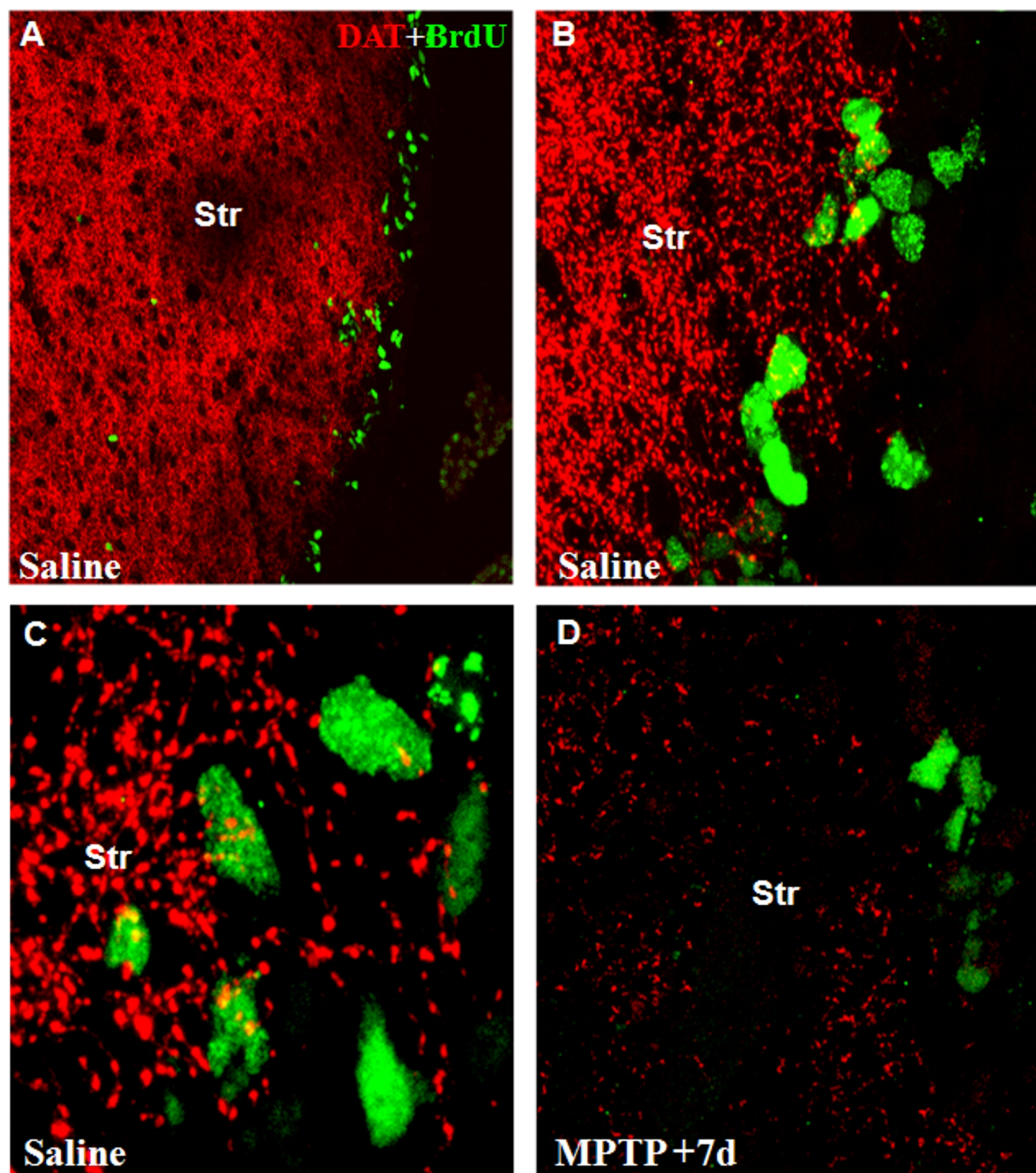

Supplementary Figure 1

Supplement: Supplementary file 1 [file ACEL-19-e13101-s001.pdf]

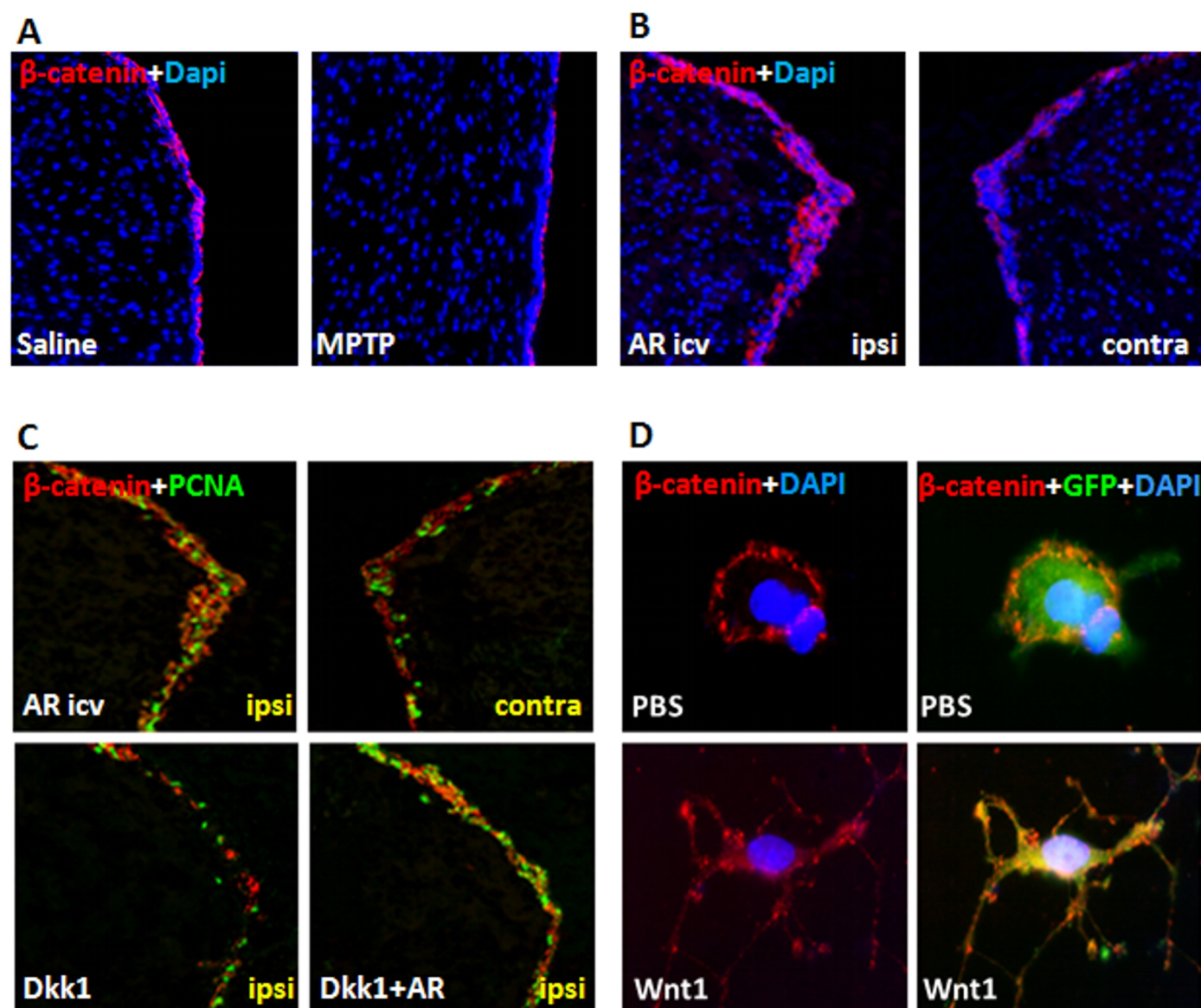

Supplementary Figure 2

Supplement: Supplementary file 2 [file ACEL-19-e13101-s002.pdf]

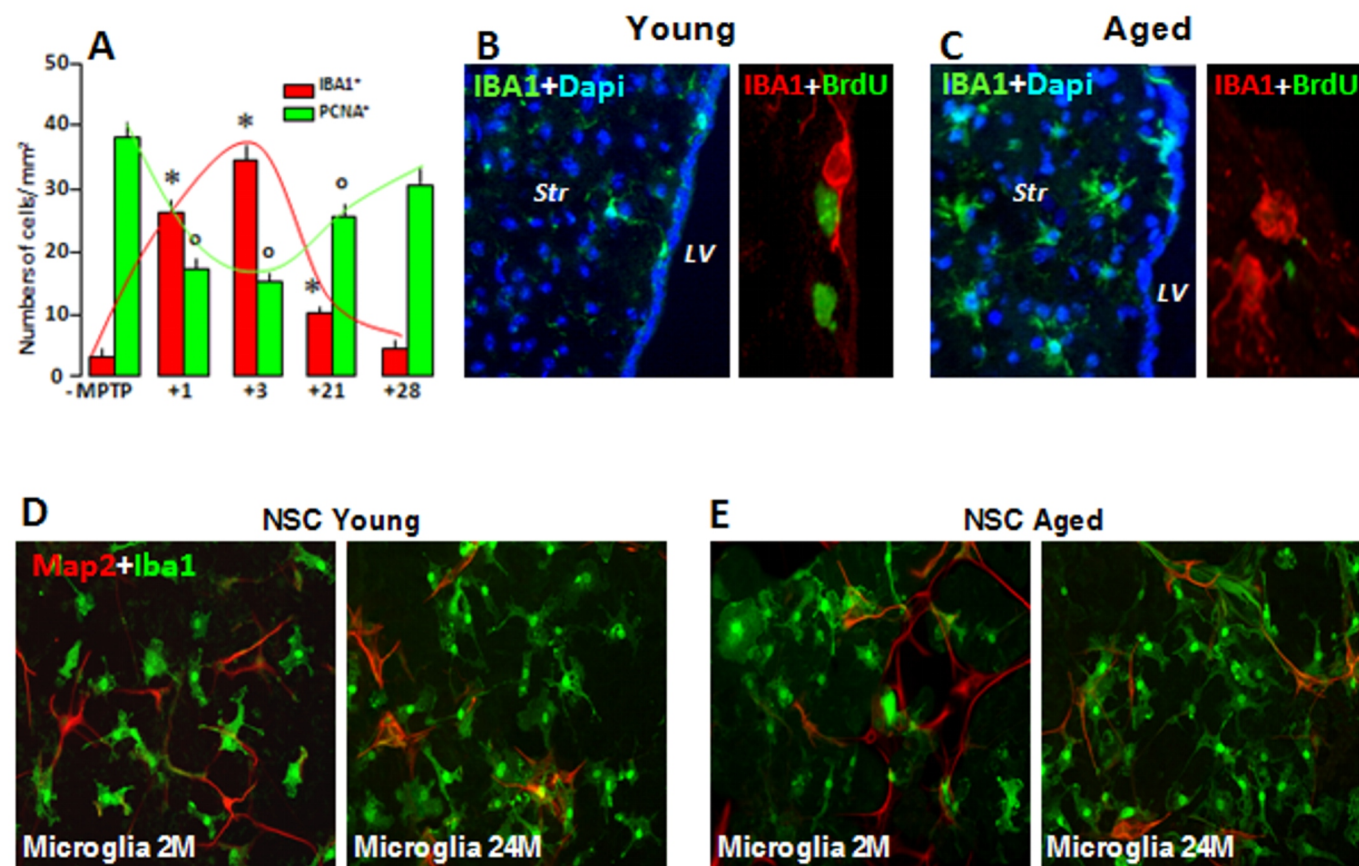

Supplementary Figure 3

Supplement: Supplementary file 3 [file ACEL-19-e13101-s003.pdf]
